# A subset of the diverse COG0523 family of putative metal chaperones is linked to zinc homeostasis in all kingdoms of life

**DOI:** 10.1186/1471-2164-10-470

**Published:** 2009-10-12

**Authors:** Crysten E Haas, Dmitry A Rodionov, Janette Kropat, Davin Malasarn, Sabeeha S Merchant, Valérie de Crécy-Lagard

**Affiliations:** 1 Department of Microbiology and Cell Science, University of Florida, Gainesville, FL, USA; 2 Burnham Institute for Medical Research, La Jolla, CA, USA; 3 Institute for Information Transmission Problems (the Kharkevich Institute), RAS, Moscow, Russia; 4 Department of Chemistry and Biochemistry and Institute for Genomics and Proteomics, University of California at Los Angeles, Los Angeles, CA, USA

## Abstract

**Background:**

COG0523 proteins are, like the nickel chaperones of the UreG family, part of the G3E family of GTPases linking them to metallocenter biosynthesis. Even though the first COG0523-encoding gene, *cobW*, was identified almost 20 years ago, little is known concerning the function of other members belonging to this ubiquitous family.

**Results:**

Based on a combination of comparative genomics, literature and phylogenetic analyses and experimental validations, the COG0523 family can be separated into at least fifteen subgroups. The CobW subgroup involved in cobalamin synthesis represents only one small sub-fraction of the family. Another, larger subgroup, is suggested to play a predominant role in the response to zinc limitation based on the presence of the corresponding COG0523-encoding genes downstream from putative Zur binding sites in many bacterial genomes. Zur binding sites in these genomes are also associated with candidate zinc-independent paralogs of zinc-dependent enzymes. Finally, the potential role of COG0523 in zinc homeostasis is not limited to Bacteria. We have predicted a link between COG0523 and regulation by zinc in Archaea and show that two COG0523 genes are induced upon zinc depletion in a eukaryotic reference organism, *Chlamydomonas reinhardtii*.

**Conclusion:**

This work lays the foundation for the pursuit by experimental methods of the specific role of COG0523 members in metal trafficking. Based on phylogeny and comparative genomics, both the metal specificity and the protein target(s) might vary from one COG0523 subgroup to another. Additionally, Zur-dependent expression of *COG0523 *and putative paralogs of zinc-dependent proteins may represent a mechanism for hierarchal zinc distribution and zinc sparing in the face of inadequate zinc nutrition.

## Background

Transition metals perform vital roles in many chemical reactions essential for life. A recent bioinformatic approach suggests Zn-, non-heme Fe- and Cu-proteins constitute 10% of bacterial and eukaryotic proteomes and 13% of archaeal proteomes [1-3]. The roles of these metals can be varied. In some oxidoreductases, for instance, iron and copper are exploited for their ability to accept or donate electrons, while in hemoglobin and hemocyanin, these metals are used for oxygen transport [4-6]. Zinc, on the other hand, serves as an electrophile or Lewis acid in many protein-catalyzed reactions. The activity of metalloproteins (many of which are essential proteins) is, consequently, strictly dependent on the presence of a metal and in most cases of a specific metal. Ensuring proper metal allocation is therefore critical for survival.

It was initially assumed that free pools of metal ions were available within the cell, such that a nascent polypeptide would acquire its cofactor solely through the metal-affinity of the chelating ligands. As discussed in several recent reviews, this picture of metal metabolism was oversimplified [7,8], as: i) the ions are chelated intracellularly by proteins and small molecule ligands, and ii) metal-binding ligands are not sufficiently selective to ensure that the proper cofactor is loaded. Since the discovery of the first copper metallochaperone, Atx1[9], numerous protein factors involved in metallocenter biosynthesis have been characterized. The mechanisms by which the cell ensures the correct metal ions are loaded into metalloproteins are just beginning to be understood.

Studies involving the maturation of Ni-urease and Ni-Fe hydrogenase have provided the most extensive picture of metallocenter biosynthesis (for a review see [10] and [11]). These two nickel-containing proteins require a suite of accessory proteins to properly insert Ni into the catalytic site (only one exception has been found to date; *Bacillus subtilis *encodes a functional urease in the absence of the canonical accessory proteins [12]). In both cases, a GTPase (UreG for urease or HypB for hydrogenase) is involved in the incorporation of the Ni cofactor. These two proteins belong to the G3E family of P-loop GTPases as defined by Leipe and colleagues [13]. Other members of this family include MeaB (ArgK), required for the activation of methylmalonyl-CoA mutase (a B_12_-dependent enzyme) [14], and COG0523, a large and diverse subfamily of proteins with poorly defined functions.

COG0523 proteins occur in all kingdoms of life, and most sequenced genomes encode one or more homologs. The first member of the COG0523 family was identified as being involved in cobalamin biosynthesis in *Pseudomonas denitrificans *and hence named CobW [15]. Other members of COG0523 include the nitrile hydratase activator, which is required for Fe-type nitrile hydratase activity [16], and YciC of *Bacillus subtilis*. Due to repression by Zur, a zinc-responsive transcription factor, *yciC *was originally assumed to code for a low-affinity zinc transporter [17-19]. Without the means to automatically distinguish between these different functions, these annotations have been propagated amongst all members of the family in sequenced genomes. Therefore, as a result of these studies, genes encoding a COG0523 protein have been automatically and arbitrarily assigned either a function in cobalamin biosynthesis, in the activation of nitrile hydratase, or as a low-affinity zinc transporter. Nevertheless, we note that each of these functions is related in the general sense to intracellular metal delivery. The diversity of the metals putatively handled by COG0523, Ni, Fe, or Zn, suggests that there might be different sub-groups identifiable within the COG0523 superfamily.

The COG0523 family is a striking example of systematic, homology-based mis-annotation. Although members are frequently annotated as having specific functions, these 'functions' are based only on homology to a few family members and are therefore suspect. The simplest way to annotate a genome is based on sequence homology to characterized genes. Sequence homology does not necessarily equate to functional identity or even similarity. Therefore, this approach to annotation is frequently inadequate as exemplified in the literature [20-22] and illustrated by the development of alternative paradigms for functional annotation [23-26].

To provide an improved annotation for the various members of this family and gain insight into the role members of this protein family may perform, we conducted an extensive comparative genomic analysis of the G3E family of P-loop GTPases and more specifically of COG0523 members. By combining phylogenetic analysis, physical clustering analysis, and regulatory site detection, we predict that the COG0523 family comprises subfamilies that have specialized and distinct functions in metal metabolism. We also hypothesize that several, but not all, of these subfamilies have a role in survival under conditions of poor zinc nutrition in both prokaryotic and eukaryotic organisms.

## Results and Discussion

### Phylogenomic analysis of COG0523 proteins as members of the G3E family of P-loop GTPases

**Figure 1:**
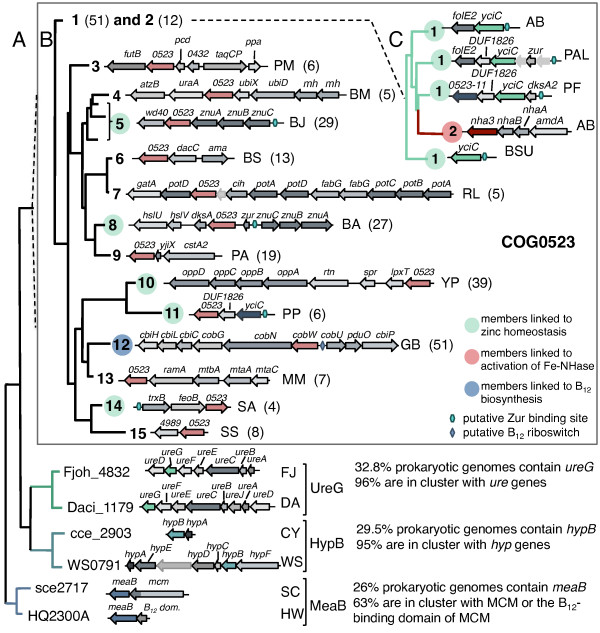
**Summary of phylogenomic analysis of G3E family and COG0523 members in prokaryotes**. A, Phylogeny of extracted GTPase domains from diverse members of the G3E family plus genome context for corresponding genes. B, COG0523 distance tree. Each subgroup (3,4 and 6-15) was collapsed to its common node. Since subgroup 5 is paraphyletic (the clade containing subgroup 5 also contains subgroup 4), branches were collapsed to three nodes. Representative gene neighborhoods for each subfamily are shown as well as corresponding genome. Numbers in parentheses refer to the number of species from which the gene cluster occurs. For subfamily 1, this number refers to the number of species that contain a putative Zur-regulated *yciC*. C, Representative branches from the subfamily 1 and 2 clade. Abbreviations: '*0523*', *COG0523 *homologs; '*yciC*', subfamily 1; '*cobW*', subfamily 12; '*nha3*', subfamily 2. For subfamily 1, '*0523-11*' refers to a subfamily 11 *COG0523 *homolog that is found in the same gene cluster as *yciC*. All other gene abbreviations and genome abbreviations can be found in Additional File 7.

**Figure 2:**
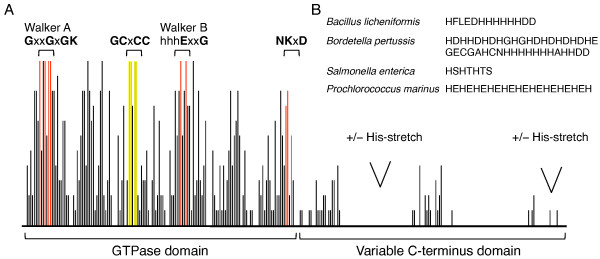
**COG0523 amino acid conservation plot**. A, Plot of amino acid conservation. The conserved GTPase motifs are highlighted in red. The conserved GCXCC motif is highlighted in yellow. The most common positions of His-stretches are shown. B, Typical histidine-rich sequence found in COG0523 homologs from specified genomes.

Phylogenetic analysis first performed by Leipe, *et al*. [13] and repeated here with a diverse set of family members (see Figure 1) shows that COG0523 belongs to the G3E family of P-loop GTPases (G3E family), which is separated from the rest of the SIMIBI class of GTPases (for SIgnal recognition GTPases, MInD superfamily, and BIoD superfamily) by a glutamate residue in the Walker B motif and an intact NKXD motif (Figure 2A) [13]. Characterized members of the G3E family perform two roles in metallocenter assembly: 1) facilitating incorporation of the cofactor in an energy- dependent manner into the target protein's catalytic site (the insertase role) and, 2) storage and delivery of a metal cofactor to a target metalloprotein (the metallochaperone role). G3E family proteins have been found to function as either metal-insertases or as a dual function metallochaperone/insertase. For example, MeaB appears to fulfill the role of an adenosylcobalamin (Co^2+^)-insertase, facilitating the insertion of B_12 _into methylmalonyl-CoA mutase (MCM) [27]. A large structural rearrangement occurs upon interaction between MeaB and its target [27], suggesting that MeaB may be responsible for the structural changes that must occur for B_12 _cofactor incorporation. In addition, MeaB also appears to protect radical intermediates that are essential for the activity of MCM [28,29]. In hydrogenase maturation, HypB is thought to carry out both the insertase and metallochaperone roles in most organisms due to the presence of a histidine stretch located at the N-terminus of these proteins. In *Bradyrhizobium japonicum*, this histidine stretch was found to bind 18-Ni ions per dimer [30]. In *Escherichia coli*, the common histidine stretch is missing and SlyD is presumed to be the metallochaperone component that delivers Ni to the assembly complex [31,32]. Most UreG proteins studied to date lack the histidine stretch that is found in most HypB proteins. Accordingly, in urease maturation, UreG appears to function as an insertase and another accessory protein, UreE, functions as the metallochaperone, delivering Ni to the maturation complex [33-35].

To support the proposition that the presence of a histidine stretch in G3E family proteins could be indicative of metallochaperone activity, we analyzed the distribution of genes encoding UreG and UreE (see 'G3E' subsystem in the SEED database [36]). We found that several genomes containing *ureG *homologs lack any recognizable *ureE *homologs. In all but two of those genomes, UreG contains an added histidine-rich motif at the N- or C-terminus (Table 1). As has been suggested for the UreG of *Mycobacterium tuberculosis*, this His-stretch may be able to compensate for the absence of UreE [37]. In addition, the absence of *ureE *does not correlate with the presence of *slyD *as would be expected if SlyD performs the metallochaperone role in those organisms. *Bradyrhizobium japonicum *USDA 110 and *Frankia *sp. Ccl3 are two exceptions to this trend as they lack both *ureE *and a His-stretch extension in UreG. In these cases, the Ni-metallochaperone involved in urease maturation could be HypB, which is present in both of these organisms (see 'G3E' subsystem). Indeed, it has been shown in *Helicobacter pylori *that HypB is required for activity of both hydrogenase and urease [38], and a physical interaction between UreG and HypB has been verified [39]. Although *ureE *is present in the genome of *H. pylori*, the corresponding protein lacks a His-stretch. As expected, the addition of a His-stretch to UreE was found to eliminate the need for HypB in the maturation of urease [40].

**Table 1:** Co-occurrence profile between *ureE, slyD*, and a His-stretch in UreG.

**Organism**	***ureE***	**UreG histidine stretch**	***slyD***
*Anaeromyxobacter *sp. Fw109-5	-	HDHSLHSGHDHGLGPGSFHDRGAPH	+
*Arabidopsis thaliana*	-	HDHHHHHHDHEHDH	-
*Bradyrhizobium japonicum *USDA 110	-	-	-
*Cytophaga hutchinsonii *ATCC 33406	-	HLDHFDSPGHFHHRELIH	+
*Frankia *sp. Ccl3	-	-	-
*Gibberellazeae *PH-1	-	HSHDGQSHSHDGFNAQEHGHSH	+
*Herpetosiphon aurantiacus *ATCC 23779	-	HVHDDHHHHHHH (C-terminus)	-
*Magnaporthe grisea *70-15	-	HSHSHDGSAPHSHSHDGSTFNAQEHGHSH	+
*Mycobacterium bovis *AF2122/97	-	HSHPHSH	-
*Mycobacterium marinum *M	-	HSHDHTHDHH	-
*Mycobacterium tuberculosis *CDC1551	-	HSHPHSH	-
*Mycobacterium vanbaaleni vanbaalenii *PYR-1	-	HFLDGQPHGH	-
*Neurospora crassa*	-	HTHSHDHGDGGHHHHPHSHSHDFNSQSGFNAQEHGHSH	+
*Nocardia farcinica *IFM 10152	-	HDHAH	-
*Schizosaccharomyces pombe*	-	HKGGSDDSTHHHTHDYDHHNHDHHGHDHHSHDSSSNSSSEAARLQFIQEHGHSH	-
*Sorangium cellulosum *So ce 56	-	HDPGEHGHGRHDHDHDHDHVHDHDHDHDHVHGGGHRHAHEHEHAHEHAHGHEHGHAHAHAHAHAHEHAHGHTHEHWAH	+
*Streptomyces avermitilis *MA-4680	-	HLDHAHTH	-
*Streptomyces coelicolor *A3	-	HLDHHH	-
*Verminephrobacter eiseniae *EF01-2	-	HHLHH	+
*Bacillus cereus *ATCC 10987	+	-	-
*Corynebacterium glutamicum *ATCC 13032	+	-	-
*Haloarcula marismortui *ATCC 43049	+	-	-
*Rhizobium leguminosarum *bv. *viciae *3841	+	-	-
*Ureaplasma urealyticum *serovar 10	+	-	-
*Helicobacter pylori *26695	+	-	+

We compared the amino acid sequence of 887 COG0523 proteins from all kingdoms (see 'G3E' subsystem for sequences). We observed that like UreG and HypB orthologs, COG0523 proteins are found with and without His-stretches, suggesting a distribution of insertase and metallochaperone activity among various members. Approximately 40% of the sequences analyzed contain a histidine-rich region, commonly found near the C-terminus (Figure 2A and 2B); 365 COG0523 proteins contain the minimal HxHxHxH motif, where x represents 0 - 4 residues. Some proteins contain a His-stretch with up to 29 histidines, such as Ava_3717 [Genbank:75703646] from *Anabaena variabilis*.

The region of highest similarity between COG0523 and the other members of the G3E family is the GTPase domain, defined by the canonical Walker A and Walker B motifs (Figure 2) [13]. GTPase activity of HypB and UreG has been shown to be essential for the metallocenter biosynthesis of hydrogenase and urease, respectively [41,42], and postulated to be responsible for incorporation of B_12 _in MCM by MeaB [29]. GTPase activity has been verified for YjiA, a COG0523 homolog from *Escherichia coli*, for which the crystal structure was solved [43]. In addition to the GTPase domain, all members of COG0523 have a conserved, putative, metal-binding CXCC motif (Figure 2A). Analysis of the YjiA crystal structure reveals this motif is found in the Switch I region of the protein, suggesting that binding of GTP/GDP affects its conformation [43]. The same motif was found to be essential for the activity of the nitrile hydratase activator protein, a member of COG0523 assumed to be involved in the incorporation of iron into Fe-type nitrile hydratase [44,45].

In addition to the GTPase domain, MeaB and most COG0523 proteins contain an additional C-terminal domain. On average, COG0523 is 99 and 147 amino acids larger than HypB and UreG, respectively, and only 26 residues larger than MeaB. The smallest G3E protein, UreG, is the GTPase component of a complex composed of UreD and UreF, where the three proteins act together in the activation of urease [46]. Activation of MCM appears to only require delivery of the cofactor by adenosyltransferase and the activity of MeaB[47]. The size of G3E proteins could be indicative of the number of other accessory proteins required for activation of the target metalloprotein.

While the N-terminal GTPase domain is well conserved among COG0523 members, the C-terminal region is highly variable (Figure 2A). Indeed, COG0523 proteins fall under the category of "segmentally variable genes (SVGs)," as defined by Zheng *et al *[48]. SVG profiles for four members of the family (HP0312, NMB1263, VCA0527, yeiR) can be found at . SVGs are genes that code for proteins that have highly variable regions interspersed with well-conserved regions. The authors observed that SVGs encode proteins that are involved in adaptation to environmental stresses and proposed that highly variable domains are an indication of protein-protein interaction specificity or specificity of small molecule binding.

Finally, as summarized in Figure 1, while *hypB *genes are consistently found in hydrogenase maturation gene clusters, *ureG *genes in the urease maturation clusters and *meaB *genes cluster with MCM-encoding genes, *COG0523 *genes are found in multiple gene clusters. Most genomes contain only one homolog of *hypB*, *ureG*, or *meaB*. Conversely, up to 11 *COG0523 *genes can be found in a single genome, as seen in *Cyanothece *sp. ATCC 51142. In addition, the available functional analyses of COG0523 members suggest varied functions and an interaction with various metals [15,18,45]. We predict that the different gene clusters involving COG0523 represent distinct subgroups. In contrast to the HypB, UreG, and MeaB subfamilies, which are composed of chaperones for a single protein, each COG0523 subgroup may perform a chaperone role in different metallocenter biosynthesis of various proteins.

In summary, this analysis suggests that like HypB or UreG, COG0523 proteins are most certainly metal insertase and/or metallochaperones. However, the metal substrate and the metalloprotein target(s) of most COG0523 family proteins is not obvious, and the initial analysis of the *COG0523 *gene neighborhoods suggests that there could be a greater diversity of targets than observed for the other G3E family subgroups. To investigate the presence of diverse COG0523 subfamilies, we combined literature analysis (Table 2 and Additional File 1) with predictions of Zur and B_12 _regulation (see below) and phylogenetic and gene neighborhood analyses. This approach led to the identification of fifteen subfamilies (summarized in Figure 1, detailed in Additional Files 2 and 3). Each subfamily is monophyletic and the corresponding genes belong to similar genomic neighborhoods and/or share conserved regulatory sites. Two exceptions are subfamilies 1 (Figure 1C) and 5 (Figure 1B), which appear to be paraphyletic; the clade composed of subfamily 1 also contains subfamily 2 and the clade that contains subfamily 5 also contains subfamily 4 (Additional File 2). Five subfamilies (1, 2, 5, 12 and 13) are analyzed in more detail below. The 10 others are detailed in Additional File 3.

**Table 2:** Literature reports of COG0523 mutant data.

**Organism**	**COG0523**	**Phenotype**	**Ref**.
*Bacillus subtilis*	YciC	EDTA-sensitivity in a Δ*ycdH *background	[17,18]
*Brucella suis*	BRA0987	Deficiency in intramacrophagic replication	[70]
*Burkholderia pseudomallei*		Inability to infect *C. elegans*	[71]
*Pseudomonas denitrificans*	CobW	Cobalamin-minus	[15]
*Saccharomyces cerevisiae*	YNR029c	Salt- and heat-sensitivity	[89]
		EGTA-sensitivity	[88]

### The CobW subfamily involved in cobalamin biosynthesis

CobW was the first member of COG0523 to be described and so-named because the disruption of the corresponding gene in *Pseudomonas denitrificans *resulted in the inability to synthesize cobalamin (Table 2) [15]. Although "cobalamin biosynthesis protein" is the most highly propagated annotation for COG0523 members, our comparative genomic and phylogenetic analysis reveals that true CobW proteins (Subgroup 12, Figure 1) represent only 12.5% of the COG0523 family (for a list of putative *cobW *genes, see 'Coenzyme B12 biosynthesis' subsystem). In our previous genomic analysis, *cobW *genes were identified in Proteobacteria located within the cobalamin biosynthesis gene clusters under control of the B_12 _riboswitch, a regulatory RNA element modulating gene expression in response to changing B_12 _concentrations [49]. As many more genome sequences have become available, we updated this analysis and report that 54 out of 65 *cobW *orthologs analyzed belong to B_12_-regulated gene clusters in γ-, β-, and α-proteobacteria (Additional File 4). In three α-proteobacteria from the Rhodospirillaceae family, *cobW *genes belong to the cobalamin biosynthesis gene clusters that are not preceded by B_12 _riboswitches. Finally, *cobW *orthologs in cyanobacteria are neither clustered with B_12 _biosynthesis genes nor regulated by a B_12 _riboswitch. However, these orthologs are highly similar to other CobW proteins and the corresponding genes co-occur with the cobalamin biosynthesis genes of the aerobic pathway.

In the majority of cases, *cobW *is found adjacent to the cobalt chelatase component, *cobN *(Figure 1 and Additional File 3) and all CobW proteins analyzed contain a His-stretch, which on average is composed of 7 histidines (the least being 4 histidines and the most being 15). The exact function of CobW is still not clear; it could be involved in the presentation of the cobalt ion to the cobalt chelatase, protection of the cofactor, or involved in inserting a metal in a metal-dependent enzyme of the pathway, such as Fe-dependent CobG [50].

### The Nitrile hydratase activator subfamily

Based on our analysis, less then 0.7% of the COG0523 family is represented by the nitrile hydratase (NHase) activators (Subgroup 2, Figure 1). A complete list of identified Fe-type NHase activators from both Genbank and SEED databases is given in Additional File 3. In the literature, these proteins are referred to as Nha3, P44K, or P47K, depending on the organism in which the protein was identified (Additional File 3). Here we refer to this subgroup of COG0523 as Nha3. Nha3 is found clustered exclusively with the genes encoding the two subunits of the Fe-type NHase (Figure 1 and Additional File 3) and has been found to be required for the *in vivo *activity of Fe-type NHase [16]. NHases are enzymes that use either a non-heme iron (III) or non-corrin cobalt (III) for the hydration of nitriles to amides [51]. NHase types can be differentiated by the strictly conserved metal binding motifs CSLCSCT for Fe(III) and CTLCSCY for Co(II) [52]. Although, the same coordination geometry has been determined for both Co(III)- and Fe(III)-binding sites [53], the two types of NHases specifically incorporate the correct metal. This specificity is thought to be due to activator proteins, which are required for full activity of their respective NHase. For Co-type NHases, metallocenter biosynthesis is thought to occur via subunit exchange, a mechanism called "self-subunit swapping" [54,55]. The accessory protein in this case, NhlE, is a self-subunit swapping chaperone and the corresponding gene is always found adjacent with the target NHase genes (Additional File 3). No sequence similarity is found between the Co-type accessory protein and the Fe-type accessory protein supporting the conclusion that Co- and Fe-type metallocenters are assembled by different mechanisms.

Even if the involvement of Nha3 in Fe-type NHase activation is documented, its exact role is not known. It has been postulated that it has an insertase role involved in incorporation of iron into the active site of the hydratase [45]. When the Fe-dependent NHase from *Rhodococcus *sp. N-771 was expressed in *E. coli *without Nha3 in Co-supplemented media, it incorporated Co instead of Fe [56]. Therefore, nitrile hydratase activator proteins may not only be involved in incorporating Fe, but also in ensuring that competing metal ions are excluded. In addition, the coexpression of Nha3 with NHase was found to be unnecessary with the coexpression of the GroESL chaperones [57]. This observation supports the hypothesis that COG0523 proteins like the rest of the G3E family could be involved in the structural rearrangements that must take place to ensure the metal cofactor is incorporated into the catalytic site.

### Zur-regulated COG0523 proteins

Extensive analysis of the literature (Table 2 and Additional File 1) reveals that members of COG0523 have been implicated in the virulence of several pathogens whose hosts are known to induce Zn-limitation as a defense strategy. In 1973, Kochan introduced the concept of nutritional immunity as a defense strategy against invading pathogens [58]. The host organism actively deprives metals from the invaders inducing both hypoferremia and hypozincemia (deficiency of iron and zinc, respectively, in the blood) as part of the acute inflammatory response [59-61]. Therefore, the mechanisms that enable a pathogen to overcome this host-induced Zn-starvation are considered essential to a pathogen's ability to cause infection [62-64]. In *Mycobacterium tuberculosis*, a *COG0523*-like gene, *RV0106*, (shown also to be repressed by Zur [65]) is up-regulated during human macrophage infection [66] (although RV0106 shows homology to COG0523, it is missing both GTPase motifs, and the second cysteine of the CXCC motif is not conserved). In the closely related *Mycobacterium avium *subsp. *paratuberculosis*, this gene is found on a pathogenicity island [66,67] and the corresponding protein was the second strongest antigen consistently reactive with cattle sera infected with *M. avium *or *Micobacterium bovis *[68]. *COG0523 *is also found in a pathogenicity island from *Enterococcus faecalis *[69]. The loss of *COG0523 *in *Brucella suis *rendered this bacterium incapable of intramacrophagic replication [70], while the loss of *COG0523 *in *Burkholderia pseudomallei *results in the inability to infect *Caenorhabditis elegans *(Table 2) [71]. An ortholog from *Francisella tularensis *was expressed exclusively in bacteria separated from infected murine spleen tissue [72]. This gene is down-regulated in the *Francisella novicida ΔpmrA *mutant [73]. PmrA is a transcription factor found to be essential for survival/growth inside human and murine macrophage cell lines [73].

In plants, an opposing defense strategy may be employed, as repression of zinc uptake machinery is required for full virulence of the plant pathogens, *Xanthomonas campestris *and *Xanthomonas oryzae *[74-76]. In contrast to animal pathogens and further supporting a role for COG0523 in zinc homeostasis, two *COG0523 *homologs of *Agrobacterium tumefaciens *as well as the genes encoding the high-affinity zinc transporter, ZnuABC, are down-regulated in response to plant signals (Additional File 1) [77].

The most extensive analysis of the zinc-dependent regulation of a *COG0523 *gene has been performed on the *COG0523 *(*yciC*) in *Bacillus subtilis *encoding a member of subfamily 1 (Figure 1 and Additional File 3). The expression of *yciC *is controlled by the Zn-dependent Zur repressor and is thus up-regulated under Zn-limiting conditions [17]. In addition to the work on *yciC*, an early comparative genomic analysis had identified Zur-regulated *yciC *orthologs in several Gram-positive bacteria (*Bacillus*, *Staphylococcus*, *Enterococcus*) [62]. As this initial analysis of putative Zur-binding sites had been done when a limited set of genomes was available and, as discussed above, scattered observation links this family to zinc limitation, we expanded the analysis to all currently complete bacterial genomes (see Methods).

Sixty-eight *yciC*/*COG0523 *genes were found to be downstream of a potential Zur-binding site mainly in Firmicutes and γ-, β-, and α-proteobacteria (Additional File 5). Two *COG0523 *genes were found downstream of a putative Zur site in the cyanobacteria, *Prochlorococcus marinus, Nostoc *sp. PCC 7120 and several *Cyanothece *species (Additional File 5). While most proteins encoded by Zur-regulated *COG0523 *members are found in subfamily 1 (75%), several paralogs are found in other subfamilies. For instance, in *Pseudomonas entomophila, Pseudomonas fluorescens*, and *Pseudomonas putida *there are two *COG0523 *homologs per genome predicted to be downstream of a Zur-binding site (Additional File 5). Our phylogenetic analysis reveals that one paralog belongs to subfamily 1 while the other belongs to subfamily 11 (Additional File 2). Zur-regulated *COG0523 *paralogs are also found in subfamily 5, 8, 10 and 14 (Additional File 2 and Additional File 5). One possibility is that the presence of several Zur-regulated COG0523 subfamilies could be indicative of more than one function of COG0523 under zinc limitation (as discussed below).

### Of the three COG0523 genes in *Acinetobacter baylyi *ADP1, only one is regulated by Zur

**Figure 3:**
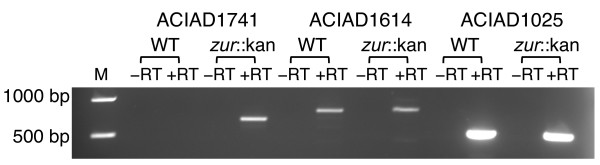
**Differential expression of the three COG0523 genes of *Acinetobacter baylyi *ADP1**. Amplification of ACIAD1741 (lane 2, 3, 4 and 5), ACIAD1614 (lane 6, 7, 8 and 9) and ACIAD1025 (lane 10, 11, 12 and 13) transcripts from *A. baylyi *ADP1 (WT) and *A*. *baylyi *Δzur::kan (*zur*::kan). Abbreviations: M, base pair marker; -RT, reaction without reverse transcriptase; +RT, reaction with reverse transcriptase.

To test the predictive power of our COG0523 phylogenomic analysis, the regulation of the three *COG0523 *genes from *Acinetobacter baylyi *ADP1 was analyzed. The first, *ACIAD1614 *[Genbank: ACIAD1614, 49530751], is predicted to be a *nha3 *homolog (subfamily 2). The second, *ACIAD1025 *[Genbank: ACIAD1025, 49530203], is predicted to be most similar to subfamilies 10 and 11. The third, *ACIAD1741 *[Genbank: ACIAD1741, 49530869], is predicted to be regulated by Zur (subfamily 1). These groupings suggest that the expression of only the ACIAD1741-encoding gene should be under Zur control. Expression of the three *A. baylyi COG0523 *genes was analyzed by RT-PCR (see Material and Methods) in a WT strain and in a *Δzur *derivative (*ΔACIAD0176*). As shown in Figure 3, the presence or absence of Zur does not affect the expression of *ACIAD1025 *and *ACIAD1614 *under the conditions tested. Nevertheless, as we predicted, *ACIAD1741 *is only expressed in *Δzur *background.

### Identification of putative Zur-regulated genes encoding paralogs of Zn-dependent enzymes

**Figure 4:**
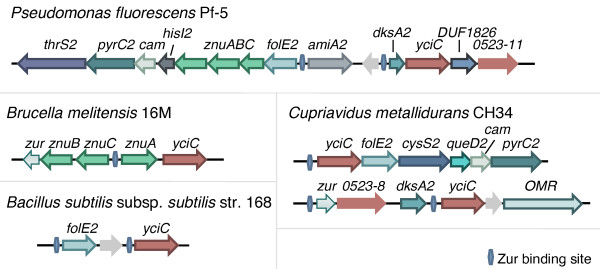
**Representative gene clusters composed of Zur-regulated *COG0523 *members**. Genes labeled *yciC *represent subfamily 1 *COG0523 *members. *COG0523-11 *and *COG0523-8 *refer to subfamilies 11 and 8, respectively. Abbreviations not found in text: DUF1826, Pfam protein family of unknown function; OMR, outer membrane protein.

**Figure 5:**
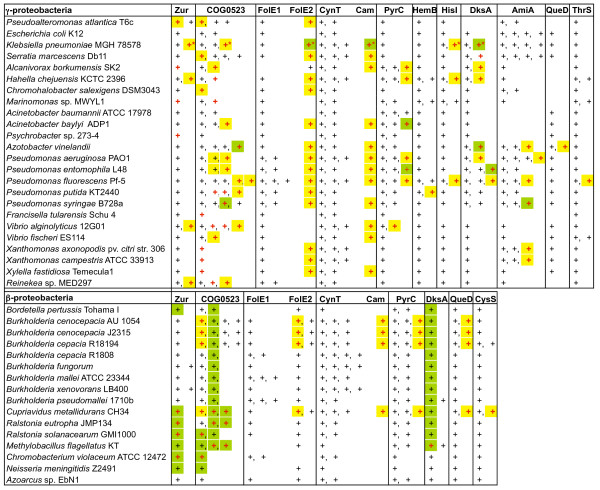
**Genomic co-localization of genes belonging to putative Zur regulon**. Genomic distribution, candidate Zur-dependent regulation and genomic co-localization of genes encoding Zur, COG0523 and Zn-dependent enzymes and their paralogs in γ- and β-proteobacteria. The presence of genes encoding the respective protein (columns) is shown by '+'. Multiple paralogs are shown by '+' separated by a comma. Genes clustered on the chromosome (e.g. operons) are highlighted by a matching color. Genes predicted to be regulated by Zur are marked in red. Zur-regulated gene cluster on the virulence plasmid, pLVPK, of *Klebsiella pneumoniae *is marked by an asterisk.

Genome context analysis revealed that a significant proportion of the *yciC *genes (Zur-regulated COG0523) are located within chromosomal gene clusters including genes for zinc transport (e.g., *znuABC*), the *zur *repressor, and various genes encoding paralogs of zinc-dependent proteins (Figure 4 and 5). Nine families of Zn-dependent enzymes whose paralogs belong to Zur regulons in γ-, and β-proteobacteria (Figure 5) were found. These Zn-dependent enzymes include phosphoribosyl-AMP cyclohydrolase (HisI), dihydroorotase (PyrC), γ-class carbonic anhydrase (Cam), porphobilinogen synthase (HemB), cysteinyl-tRNA synthetase (CysRS), threonyl-tRNA synthetase (ThrRS), N-acetylmuramoyl-L-alanine amidase, queuosine biosynthesis enzyme QueD, and C4-type zinc finger regulator DksA (see 'Zinc regulated enzymes' subsystem).

Differential regulation of distinct isofunctional genes by co-factor availability is a known regulatory mechanism in bacteria (for a review, see [78]) and in eukaryotes (as discussed below). For instance, the coenzyme B_12_-independent isozymes of methionine synthase and ribonucleotide reductase are regulated by B_12 _riboswitches in the genomes that encode both B_12_-dependent and -independent isozymes [49]. Likewise, a similar regulatory strategy has been described for zinc availability. Zn-independent proteins are negatively regulated by Zur and expressed under Zn-limiting conditions to replace the Zn-dependent proteins. Examples include paralogs of ribosomal proteins [62] and alternative isozymes of GTP cyclohydrolase I (FolE1 and FolE2) [79]. In both cases, a Zn-dependent protein is functionally replaced by a Zn-independent counterpart during conditions of zinc deficiency.

Our comparative analysis of Zur regulons revealed co-regulation and frequent co-localization on the chromosome between *COG0523* and paralogs of these Zn-dependent enzymes. For example, *Cupriavidus metallidurans *has a Zur-regulated gene cluster encoding YciC, FolE2, and paralogs of CysS, QueD, Cam, and PyrC, whereas the Zur regulon in *Pseudomonas fluorescens *includes two COG0523 proteins, FolE2, and paralogs of AmiA, DksA, HisI, Cam, and PyrC (Figure 4 and 5).

We hypothesize that these alternative enzymes could require a metal other than Zn (or no metal) and are therefore expressed during Zn-limitation to replace or compensate for the decreased activity of their Zn-dependent counterparts. Indeed, the carbonic anhydrases found in our analysis are members of the γ-class. The γ-class carbonic anhydrase from *Methanosarcina thermophila *exhibited highest activity with Fe and, when purified under anaerobic conditions, contained Fe and not Zn [80,81]. The Zur-regulated *cam *we have identified could therefore encode an Fe-dependent carbonic anhydrase expressed to compensate for the Zn-dependent carbonic anhydrases.

The proteins of three other families downstream of putative Zur binding sites are missing the conserved zinc binding residues. As shown in Figure 5 and Additional File 6, some genomes encode three PyrC paralogs. One paralog is similar to the dihydroorotase from *Escherichia coli*. These proteins have a binuclear zinc center chelated by the conserved metal binding residues His 16, His 18, Lys 102, Asp 250, His 139 and His 177 [82]. The second PyrC paralog is an inactive dihydroorotase, which is referred to in the literature as PyrC' [83]. Similar to PyrC', the zinc-binding residues are not conserved in the PyrC paralog whose gene we predict to be regulated by Zur. However, unlike PyrC', this PyrC paralog has previously been shown to display dihydroorotase activity [84]. For porphobilinogen synthase, the existence of zinc binding and non-zinc binding variants is documented in the literature [85]. As expected, HemB1 contains the zinc chelating cysteine ligands while those cysteines are not conserved in the protein, HemB2, encoded by the gene putatively regulated by Zur in *Pseudomonas putida *(Additional File 6). HemB2 we would accordingly expect to be active with magnesium and/or potassium instead of zinc. In addition, the DksA paralogs downstream of putative Zur binding sites are missing the canonical C4-zinc finger motifs (Additional File 6).

Not all paralogs seem to have lost their zinc-binding sites as the zinc-binding residues are conserved in the HisI, CysRS, ThrRS, QueD, and AmiA paralogs encoded by genes predicted to be induced during zinc depletion. An alternative explanation for the existence of these paralogs could be to increase protein copy number during zinc deficiency. The analysis of the metal content of some paralogs identified in this study is currently underway.

### COG0523 in eukaryotes: Two *Chlamydomonas reinhardtii *COG0523 homologs are induced under zinc limitation

COG0523 is widespread in eukaryotes, with most organisms containing one to four homologs (see 'G3E'subsystem), and have been associated with stress phenotypes (see Table 2 and Additional File 1). In *Arabidopsis thaliana*, one of the three COG0523 genes (*At1 g80480 *[Genbank: AT1g80480, 51536562]), which was isolated as a member of the actively-transcribed plastid chromosome in mustard seed [86], is induced under heat-stress [87]. Deletion of *COG0523 *from *Saccharomyces cerevisiae*, *YNR029c *[Genbank: 6324356], confers sensitivity to the metal chelator, glycol-bis (2-aminoethylether)-N,N,N',N'-tetraacetic acid (EGTA) [88] as well as salt-sensitive and heat-sensitive phenotypes [89] (Table 2).

**Figure 6:**
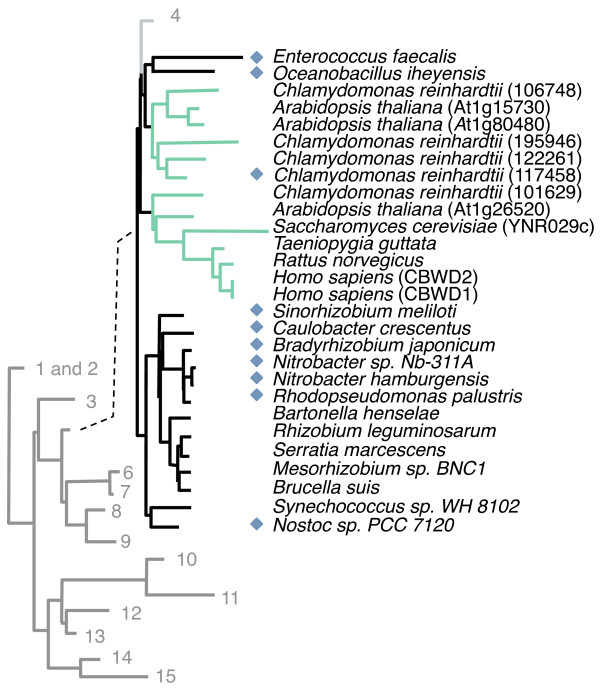
**Phylogeny of eukaryotic COG0523 members**. Lightly shaded tree represents collapsed COG0523 tree. Branches are labeled with corresponding subfamily number. Subfamily 5 Tree: branches representing eukaryotic homologs are colored. Blue diamonds indicate putative Zur-binding sites upstream of corresponding genes. For the *C. reinhardtii *ortholog, the blue diamond indicates confirmed induction of corresponding gene to zinc deficiency.

Gene clustering is not very informative in eukaryotes but most eukaryotic COG0523 homologs including *Homo sapiens *belong to subfamily 5 (Figure 6). The prokaryotic members of subfamily 5 cluster on the genome with genes that encode WD40-repeat proteins, which form a β-propeller structure thought to mediate protein-protein interactions [90], and with *znuABC *and creatinase encoding genes (Additional File 3). Several prokaryotic members of subfamily 5 are also predicted to be downstream of a Zur-binding site (Additional File 5), suggesting a role for bacterial members of subfamily 5 in the response to zinc limitation.

Little if any work has been performed on the role of COG0523 in eukaryotes, which do not encode a Zur homolog. Therefore, we sought to investigate the regulation of COG0523 during metal depletion in a eukaryotic reference organism. Previous studies have established the alga *Chlamydomonas reinhardtii *as a choice reference organism for the study of trace metal homeostasis because it is straightforward to deplete the medium of zinc, copper, iron or manganese (as seen in [91-93]). Sentinel genes for each of these deficiencies are known, such as *CYC6 *for copper deficiency, *FOX1 *for iron-deficiency, *NRAMP1 *for manganese deficiency and *ZRT3 *for Zn-deficiency [92,94,95]. Furthermore, *Chlamydomonas *has retained many pathways present in the common ancestor to the plant and animal lineages and displays the metabolic flexibility of "back up" or alternate systems [96,97]. For instance, the replacement of B_12_-independent methionine synthase with a B_12_-dependent form when this cofactor is available, the use of Mn-dependent superoxide dismutase (SOD) in place of Fe-SOD in iron-limitation and the replacement of plastocyanin with cytochrome *c*_6 _in copper-deficiency [93,98,99].

**Figure 7:**
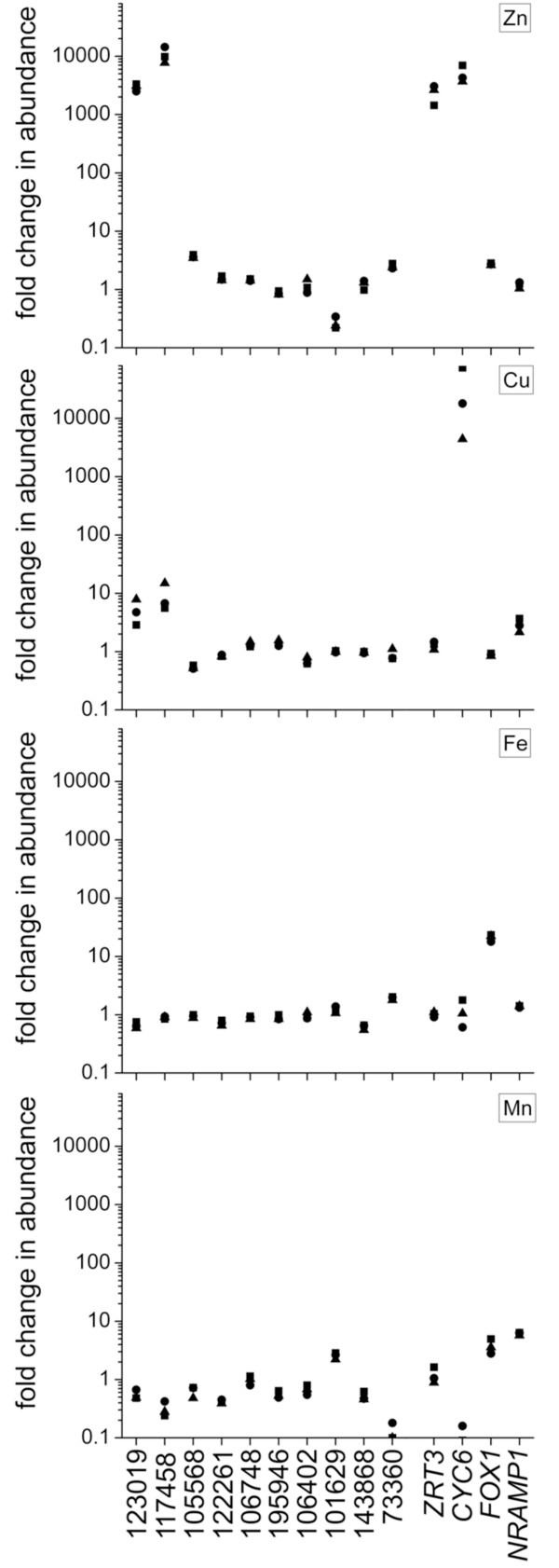
**Differential expression of the *COG0523 *genes of *Chlamydomonas reinhardtii***. *Chlamydomonas *strain 2137 was grown under various metal-deficient or replete conditions in triplicate experiments, represented by squares, circles and triangles. RNA was isolated from these cultures and analyzed by real time PCR. Each metal deficiency, zinc, copper, iron, and manganese, is shown in a separate panel. RNA abundance is expressed relative to the metal-replete condition. Each data point represents an independent experiment with each measurement representing the average of technical triplicates. *CBLP *was used as the reference gene. *ZRT3*, *CYC6*, *FOX1 *and *NRAMP1 *served as positive controls for Zn-, Cu-, Fe-, and Mn-deficiency, respectively. (Note that Cu-deficiency is a secondary effect of Zn-deficiency (Malasarn, unpublished) and Fe-deficiency is a secondary effect of Mn-deficiency [92].)

We identified 15 genes encoding proteins with COG0523 domains in versions 3.1 and 4.0 of the *C. reinhardtii *draft genome [96] (see Additional File 7 for protein IDs). Of these 15 gene models, only 10 gene models encoded full-length COG0523 GTPase domains. Therefore, we tested the expression of these 10 genes as a function of Zn, Cu, Fe and Mn nutrition. Transcripts for two of these, encoding proteins 123019 and 117458 (version 3.1 protein IDs), are increased in abundance by several orders of magnitude when cells are grown in zinc-limiting conditions as opposed to zinc-replete conditions, only slightly induced under copper limitation relative to copper-replete conditions and unaffected by iron or manganese nutrition (Figure 7). The zinc sensors and regulatory factors responsible for mediating this response to zinc depletion ARE yet unknown in *C. reinhardtii*.

Our phylogenetic analysis reveals that protein 123019 belongs to subfamily 1, while protein 117458 belongs to subfamily 5 (Figure 6 and Additional File 2). We therefore substantiate the role of COG0523 family members in Zn homeostasis in eukaryotes as well as in bacteria. Several COG0523 proteins encoded by eukaryotic genomes belong to subfamily 5 (Figure 6). We predict that the expression of some of these other eukaryotic COG0523 proteins may also be regulated by zinc.

In addition, supporting the functional diversity revealed by our gene neighborhood analysis, the expression of the eight other COG0523 family members from *C. reinhardtii *are not significantly affected by the deficiency of metals tested.

### COG0523 in Archaea

Although COG0523 was previously assumed to be missing from Archaea [13], the availability of recently sequenced genomes reveals that out of 44 archaeal genomes in the SEED database, eight genomes contain at least one *COG0523 *homolog, with *Methanosarcina acetivorans *C2A containing eight homologs. Most Archaeal members belong to subfamily 13, members of which co-localize with corrinoid-dependent methyltransferases (Figure 1 and Additional File 3). In *Methanosarcina barkeri, Methanosarcina acetivorans, Methanosarcina mazei*, and *Methanococcus maripaludis *S2, *COG0523 *clusters with genes involved in methanol:CoM methylation: *mtaA, mtaB*, (both are Zn-dependent [100]), *mtaC *(corrinoid protein [101]) and *ramM *(iron-sulfur protein [102]) (Figure 1 and Additional File 3). Clustering between *COG0523 *and methanogenesis genes is not limited to Archaea but also found in *Clostridium botulinum *(Additional File 3). Another clostridium, *Desulfitobacterium hafniense *DCB-2, encodes a COG0523 that clusters with a MeTr homolog (methyltetrahydrofolate:corrinoid/iron-sulfur protein methyltransferase) (Additional File 3). Also, proteome analysis of acetate- and methanol-grown *M. acetivoran s*cells revealed the presence of MA4382 (COG0523) in methanol-grown cells [103]. Finally, *MM1072 *(*M. mazei COG0523) *is induced to the same extent as its neighboring *ramM *homolog, *MM1071*, during growth in high salt conditions (2.38 and 2.21 fold, respectively) [104].

Archaeal genomes sequenced to date lack any recognizable homolog of the Fur (Fe) or Zur (Zn) transcriptional regulators. Alternatively, there is a large group of MntR/DtxR-like regulators in Archaea (*Methanosarcina, Pyrococci, Archaeaglobus, Methanobacterium*) that regulate iron homeostasis, whereas another small group of MntR/DtxR-like repressors in *Methanosarcina *spp. named ZntR (e.g. MA0022 in *M. acetivorans*), is predicted to regulate the zinc uptake operon, *znuABC *(D.A.R., unpublished observation, see Additional File 8). Comparative genomic analysis of this novel zinc regulon in *M. acetivorans *reveals that a *COG0523 *homolog, *MA4381*, is co-regulated with *znuABC *based on the presence of candidate-binding sites of the ZntR repressor (Additional File 8).

## Conclusion

### COG0523 is a diverse family of metal chaperones

Based on relatedness to the G3E family of GTPases, we expect COG0523 to also be involved in metallocenter biosynthesis of target metalloproteins. The diversity of genomic co-localization suggests that COG0523 is more diverse than the other subfamilies of G3E. Both the metal specificity and the protein target(s) might vary from one subgroup to another.

While the known roles in cobalamin biosynthesis and response to zinc limitation predominate, our analysis implies members of COG0523 are not limited to those roles. Based on genome context (co-localization and/or presence of a B_12 _riboswitch) and protein similarity analyses, only 12.5% of sequenced COG0523 from the SEED are true CobW proteins and assigned to the cobalamin biosynthesis pathway. Only ~30% of COG0523 members analyzed are linked to the zinc homeostasis either through putative Zur sites (~8%) or co-localization with genes involved in the response to zinc starvation (~20%). In addition the third known role, NHase activator, only applies to less than 1% of sequenced *COG0523 *genes. Over half of COG0523 may perform a role in the activity of unknown proteins.

### A ubiquitous subset of COG0523 is linked to zinc

Although involvement in the response to zinc deficiency applies to only a subset of COG0523, we show that this function is not limited to Bacteria but also present in Archaea and Eukaryota. Two *C. reinhardtii COG0523 *homologs, which belong to separate phylogenetic subgroups, are induced under zinc-deficient conditions compared to zinc-replete conditions. In addition, the expression of the eight other homologs was not significantly affected by metal deprivation confirming the diversity of COG0523.

### Identification of novel zinc homeostasis mechanisms

The comparative genomic analysis of the zinc repressor Zur regulons in Bacteria has revealed insights into previously unknown zinc homeostasis mechanisms. At least nine protein families that are homologs or isozymes of known Zn-dependent proteins were identified as candidate members of the reconstructed Zur regulons in γ- and β-proteobacteria, suggesting their up-regulation during zinc limitation. Based on sequence analysis, four of these protein families do not contain the canonical zinc binding residues. We propose, therefore, that these paralogs may require a metal other than Zn for catalysis and are involved in the adaptation to poor zinc nutrition. The presence of these paralogs could aid in compensating for the loss in activity of the Zn-dependent protein analogs and reducing in the total amount of Zn required by the cell.

### Putative roles of COG0523 in response to zinc limitation

At this stage, if the exact role of COG0523 members in survival in low zinc conditions is still to be determined, several hypotheses can be proposed. Our comparative genomic analysis suggests that COG0523 may be a metal chaperone for a protein that is also part of the Zur regulon. The 'alternative enzymes' of Zn-dependent proteins may require a metal other than zinc for catalysis and may also require a metal chaperone for efficient cofactor acquisition. However, in about half of the genomes analyzed, *yciC *(Zur-regulated *COG0523*) appears to not belong to any operon. For instance, although adjacent to *folE2*, *yciC *is usually regulated by Zur independently (Figure 5).

Another possibility would be that COG0523 is involved in the allocation and reallocation of zinc. Zinc is not an essential cofactor for metabolic steps where zinc-independent back-up proteins can substitute. Accordingly, in conditions of poor zinc nutrition, we expect that zinc delivery is prioritized to proteins that do not have zinc-independent substitutes (and where zinc function is hence essential). Induction of the *C. reinhardtii *genes, 123019 and 117458, containing the putative metal delivery COG0523 domain, may affect prioritized delivery to a subset of zinc targets. These delivery factors might be particularly important in a compartmentalized eukaryotic cell. In bacteria, COG0523 may also function as either a zinc chaperone as proposed recently by Gabriel *et al*. [105] or as a molecular chaperone that aids in the folding of essential zinc metalloproteins ensuring that essential Zn-proteins acquire zinc while nonessential proteins are excluded (the possible existence of a zinc metallochaperone is discussed in the recent review [106]).

As a third hypothesis, some members of COG0523 may be a chaperone involved in incorporating a metal other than zinc into Zn-dependent enzyme(s) based on zinc availability. *In vitro*, the activity of several Zn-dependent enzymes is slightly less, the same, or in some cases higher with a metal cofactor other than zinc (for recent examples see [107-109]). Under zinc limitation and supplementation with cobalt, the zinc in carbonic anhydrase of the marine diatom, *Thalassiosira weissflogii*, is substituted with cobalt *in vivo *[110]. The genome of the closely related *Thalassiosira pseudonana *encodes seven COG0523 proteins. Interestingly, the genomes of cyanobacteria and algae tend to encode relatively high numbers of COG0523 proteins. Zinc-containing carbonic anhydrases are important for assimilation of CO_2_, and algae tend to express multiple isoforms in various organelles [111], which might require mechanisms for preferential metal delivery. The symbiotic alga *Chlorella *sp. NC64A has twelve homologs and the free-living *Chlorella vulgaris *C-169 has seven. The genome of *Micromonas *sp. RCC299 encodes ten COG0523 homologs and the smallest known free-living eukaryote, *Ostreococcus tauri*, has four homologs. As stated above, the cyanobacterium *Cyanothece *sp. ATCC 51142 has eleven homologs, while *Anabaena variabilis *has five and *Nostoc *sp. PCC7120 and *Prochlorococcus marinus *susp. *marinus *both have four homologs. This high number of paralogs might reflect their particular lifestyles.

Lastly, there is some evidence from *Magnetospirillum magneticum *AMB-1 that a MeaB homolog may function as a cytoplasmic ATPase required for energizing Fe uptake [112]. Indeed, neither MeaB homolog encoded in the *M. magneticum *genome appears to be co-transcribed with a gene encoding methylmalonyl-CoA mutase (also the case for ~40% of *meaB *homologs; see 'G3E' subsystem). Therefore, a role for some COG0523 members in affecting metal transport cannot be ruled out at this point.

Further experimental work is now required to discriminate between these different potential roles. To complicate the problem, there are up to three Zur-regulated *COG0523 *paralogs in some genomes, therefore, a combination of the above functional hypotheses may prove to be operational.

## Methods

### Comparative genomic analysis of COG0523 gene family and G3E family

Analysis of 'COG0523,' 'G3E,' and 'Zinc regulated enzymes' subsystems were performed in the SEED database [23,36]. *COG0523 *gene sequences in the SEED database were identified by homology to known *COG0523 *members and the presence of the conserved CXCC motif and P-loop GTPase domain in the corresponding protein sequences. *cobW *gene sequences were identified based on homology to *cobW *from *Pseudomonas denitrificans *[15] and occurrence within cobalamin biosynthesis operons and/or downstream of a putative B_12 _riboswitch. Genomic search for candidate B_12 _riboswitches was performed as previously described [49].

### Sequence Analysis

All COG0523, HypB, UreG, and MeaB sequences presented here were downloaded from the SEED or Genbank [113] databases. The fig numbers (internal identifications in the SEED) and Genbank accession numbers can be found in Additional File 7. Identification of histidine motifs was performed with Fuzzpro from the EMBOSS software package [114]. Amino acid sequences were aligned using the ClustalW2 algorithm with default parameters [115]. For alignments of PyrC, DksA and HemB, ESPript 2.2 was also used [116]. Phylogenetic analyses were carried out by employing the Phylip 3.67 program package [117]. Distance-based matrices were generated between all pairs of sequences using the Jones-Taylor-Thornton matrix as employed in Protdist (Phylip). Phylogenetic trees were generated from these matrices using the neighbor-joining method as implemented in Neighbor (Phylip). Reliability of branches was determined with the bootstrap method of 1000 replicates using Bootseq (Phylip).

For the G3E family distance tree, the GTPase domain was extracted and aligned. The GTPase domain of CooC was used as an outgroup. Although it has been previously assumed that CooC is a member of the G3E family [14,29], GTPase sequence motifs suggest that it is actually a member of the closely related MinD/BioB family. The COG0523 distance tree was built with 177 full-length COG0523 sequences. Significant gene clusters between prokaryotic COG0523 genes and neighboring genes were identified in the SEED database. Members of COG0523 with the highest functional coupling score for each significant gene cluster were chosen for inclusion in the phylogenetic analysis. Functional coupling scores and significant gene clusters were computed by the SEED database. For an explanation of functional coupling scores refer to [118]. In addition, COG0523 proteins whose genes were identified through our analysis of the Zur regulon were also included. The COG0523 proteins from six eukaryotes were also used including the 10 COG0523 homologs from *C. reinhardtii *whose transcript levels were investigated by real-time PCR. RV0106 from *Mycobacterium tuberculosis *CDC1551 was used as an outgroup in this analysis. This protein, while having similarity to COG0523, does not contain the canonical CXCC motif (CXSC). In addition, it is missing the canonical Walker A motif of the GTPase domain, suggesting that these COG0523-like proteins do not have GTPase activity. Subfamilies were defined based on the following criteria. Each subfamily had to be monophyletic. The exceptions are subfamilies 1 and 5, which are paraphyletic. Subfamily 1 becomes monophyletic with the subtraction of the nitrile hydratase activators. Subfamily 5 becomes monophyletic with the subtraction of subfamily 4. The genes encoding proteins in each subfamily belong to similar gene clusters and/or have shared regulatory sites. Bootstrap values were all above 900. Tree illustration was performed with Treedyn [119].

### Plot of amino acid conservation

Thirty-two protein sequences representing the 15 COG0523 subfamilies were aligned using ClustalW2 and default parameters. Accession numbers for sequences used can be found in Additional File 7. Columns containing gaps in eight or more sequences were removed. Residue conservation at each position as determined by Jalview was plotted [120].

### Comparative genomic analysis of Zur regulons

Complete bacterial genomes were downloaded from GenBank [113]. The taxon-specific training sets for identification of Zur-binding motifs were composed of the previously identified Zur-binding sites in Firmicutes, α-, β-, and γ-proteobacteria [62] and the DNA motif search profiles were constructed using the SignalX program. Analyzed genomes encoding both an ortholog of Zur regulator and COG0523 proteins were scanned with the constructed taxon-specific Zur motif profiles (see Additional File 9 for sequence logo motifs) using the Genome Explorer software and the identified genes with candidate Zur-binding sites were analyzed by the consistency check comparative procedure as previously described [78]. Candidate ZntR-binding motif was obtained by applying the SignalX program to the training set of the *znuACB *regulatory regions from methonogenic Archaea that have an ortholog of the DtxR-like regulator ZntR (MA0022). Positional nucleotide weights in the recognition profile and *Z *scores of candidate sites were calculated as the sum of the respective positional nucleotide weights as described in [121]. The threshold for the site search was defined as the lowest score observed in the training set. Sequence logos for DNA-binding sites were constructed using WebLogo 2.0 [122].

### *Acinetobacter *RT-PCR

*Acinetobacter baylyi *ADP1 (ADP1) *Δzur:kan*^*R*^ was a generous gift from Véronique de Bernardinis (Genoscope, Institut de Génomique (CEA), Evry, France) [123]. Overnight cultures of ADP1 and *Δzur:kan*^*R *^cultured in Luria Broth was used to inoculate 5 ml culture of Luria Broth supplemented with 50 μM ZnSO_4 _to ensure repression of transcription by Zur. Samples (1 ml) were harvested in early stationary phase, RNAprotect Bacteria Reagent (Qiagen) was added and cells were frozen at negative 80°C overnight. Pellets were thawed and RNA was extracted using TRIzol LS reagent (Invitrogen) followed by RNeasy mini kit (Qiagen). Contaminating DNA was removed using DNase I (RNA-free) (Ambion). RT-PCR reactions were carried out with Superscript™ III One-Step RT-PCR System with Platinum^®^*Taq *High Fidelity (Invitrogen). Reactions were composed of 7.5 μl 2× reaction mix, 1 μl RNA (200 pg RNA), 0.3 μl Forward Primer (10 μM), 0.3 μl Reverse Primer (10 μM), 0.3 μl Superscript™ III RT/Platinum^® ^*Taq *High Fidelity enzyme mix, and water to a final volume of 15 μl. Reverse transcriptase minus controls were performed using 5 PRIME *Taq *Master Mix (Fisher). Reactions were composed of 6 μl 5 PRIME Master Mix, 1 μl RNA (200 pg RNA), 0.3 μl Forward Primer (10 μM), 0.3 μl Reverse Primer (10 μM), and water to a final volume of 15 μl. Growth of strains and RT-PCR were performed in experimental triplicate. Primer sequences used in this analysis are available in Additional File 10.

### *Chlamydomonas *RNA analysis

Cultures of *Chlamydomonas reinhardtii *wild-type strain 2137 were maintained in aerated Tris-Acetate-Phosphate (TAP) medium with shaking in the light (60-100 μmol m^-2 ^s^-1^). To characterize Zn-responsive gene expression, cells were initially grown to late exponential phase in TAP supplemented with 2.5 μM zinc, followed by a round of growth with no supplemental zinc, before they were inoculated into the experimental conditions at a density of 10^5 ^cells/mL. Characterization of the effects of copper, iron, and manganese was performed similarly with the exception that cultures were grown under a second round of metal deficiency prior to the experiment. The iron deficient concentration used was 1 μM [95]. When cultures reached mid- to late-exponential phase, total RNA was prepared as described in [124]. cDNA preparation and real-time PCR were performed as described in [92]with *CBLP *used as the reference gene. All experiments were performed in experimental triplicate. Additionally, all RT-PCR analysis was performed in technical triplicate. Primer sequences are available in Additional File 10. MIQE checklist is available in Additional File 11.

## List of abbreviations

MCM: methylmalonyl-CoAmutase; SVG: segmentally variable gene; NHase: nitrile hydratase.

## Authors' contributions

CEH carried out and designed the phylogenetic, comparative genomic, and sequence analysis of COG0523 and the G3E family, the RT-PCR analysis in *A. baylyi*, the sequence analysis of the Zur-regulated back-up proteins, the literature search and drafted the manuscript. DAR carried out the analysis of putative Zur and ZntR binding sites and B_12 _riboswitches, analysis of putative back-up enzyme paralogs, and assisted in drafting the paper. JK prepared *C. reinhardtii *RNA from Cu, Fe, Mn and Zn-deficient cells, designed and tested primers for qRT-PCR, performed the qRT- PCR and assisted in drafting the paper. JK and DM established conditions for zinc-deficiency in *Chlamydomonas*, isolated RNA, designed primers for qRT-PCR, and assisted in drafting the paper. SSM and VDC designed experiments, analyzed data and assisted in drafting the paper. All authors read and approved the final manuscript.

## Supplementary Material

Additional file 1 **Literature reports of COG0523 expression data.** Click here for file

Additional file 2 **Phylogeny of COG0523 subgroups**. A, Each identified subgroup is shaded and labeled. The branches representing proteins encoded by putative Zur-regulated genes are marked with a black square. The branches representing *C. reinhardtii *COG0523 homologs encoded by the genes induced by zinc deficiency are marked with a green square. Branches representing the *Pseudomonas *paralogs discussed in the text are labeled. Protein IDs for each branch can be found in Additional File 7. Click here for file

Additional file 3 **Detailed description of each COG0523 subfamily**. For subfamilies 2-15, representative genomic context figures are given as is a list of the locus-tags for members of each subfamily identified by physical clustering to common genes. Click here for file

Additional file 4 ***cobW *genes downstream of a putative B_12 _riboswitch**. Presence/absence of a putative B_12_riboswitch upstream of *cobW *is shown as well as the first gene in each putative cobalamin-regulated biosynthesis operon. CobW locus tags are in bold. B_12_riboswitches previously reported by [49] are marked with an asterisk. Click here for file

Additional file 5 **Putative Zur-binding sites in bacterial genomes**. Candidate Zur-binding sites upstream of genes encoding COG0523 family proteins and paralogs of Zn-dependent enzymes in Bacilli, α-, β- and γ-proteobacteria and Cyanobacteria. Distance refers to the location of the putative Zur-binding site upstream from the first gene in each operon. Click here for file

Additional file 6 **Sequence analysis of PyrC, DksA and HemB paralogs**. Alignments of protein sequences encoded by genomes from Figure 4. A, Alignment of the three PyrC paralogs. The residues that chelate the α-metal are highlighted in blue while the β-metal ligands are highlighted in green. Lys102 serves as a ligand for both metals ions. B, DksA paralogs. C, HemB paralogs. Secondary structure as determined from the crystal structure of the *Escherichia coli *homolog in each case is given (PDB identifiers: 1J79 (PyrC), 1TJL (DksA), 1L6S (HemB)). For PyrC and DksA, the alignments show only the portion of the alignment containing the zinc binding residues. Columns containing the zinc chelating residues as determined from the crystal structure are highlighted in yellow. Genome abbreviations: EC, *Escherichia coli*; PE, *Pseudomonas entomophila *L48; PF, *Pseudomonas fluorescens *Pf-5; PA, *Pseudomonas aeruginosa *PAO1; ABa, *Acinetobacter baylyi *ADP1; VA, *Vibrio alginolyticus *12G01; HC, *Hahella chejuensis *KCTC 2396; BCe, *Burkholderia cenocepacia *AU 1054; BC, *Burkholderia cepacia *R18194; CM, *Cupriavidus metallidurans *CH34l; AB, *Alcanivorax borkumensis *SK2; KP, *Klebsiella pneumoniae *MGH 78578 (on virulence plasmid pLVPK); AV, *Azotobacter vinelandii*; BPe, *Bordetella pertussis *Tohama I; BF, *Burkholderia fungorum*; BM, *Burkholderia mallei *ATCC 23344; BX, *Burkholderia xenovorans *LB400; BP, *Burkholderia pseudomallei *1710b; RE, *Ralstonia eutropha *JMP134; RS, *Ralstonia solanacearum *GMI1000; MF, *Methylobacillus flagelatus *KT. Click here for file

Additional file 7 **Protein IDs used in sequence analyses**. Protein IDs used for various sequence analyses are organized by figure and correspond to the indicated database. Click here for file

Additional file 8 **Putative ZntR-regulated genes in archaeal genomes**. Candidate ZntR-binding sites upstream of genes encoding COG0523, ZntR and ZnuABC in Archaea. Click here for file

Additional file 9 **Sequence logos for DNA-binding motifs for candidate Zinc regulators**. The taxonomy-specific DNA motif logos were constructed using Zur- and ZntR-binding sites indentified for *COG0523 *and other zinc-responsive genes described in the Additional Files 5 and 7. Click here for file

Additional file 10 **Primers used in transcription analyses**. Sequences for primers used in the transcription analysis of *A. baylyi *and *C. reinhardtii COG0523 *homologs. Click here for file

Additional file 11 MIQE checklist for *C. reinhardtii *qRT-PCR. Click here for file
