# Supplementary material for: A subset of the diverse COG0523 family of putative metal chaperones is linked to zinc homeostasis in all kingdoms of life
Source: BMC Genomics. 2009 Oct 12;10:470. doi: 10.1186/1471-2164-10-470 (PMC2770081; doi:10.1186/1471-2164-10-470)
Supplement: Additional file 1 — Literature reports of COG0523 expression data. [file 1471-2164-10-470-S1.PDF]

Additional File 1. Literature reports of *COG0523* expression data

Organism	*COG0523*	Gene regulation	Experimental Method	Ref.
Archaea				
Methanosarcina acetivorans	*MA4382*	Expressed during growth on methanol but not detected during growth on acetate	2D-GE/ MALDI–MS/ MS/MS	[1]
Methanosarcina mazei Gö1	*MM1072*	Up-regulated in NaCl-adapted cells versus -unadapted	Microarray	[2]
		Up-regulated during growth on methanol versus acetate	Microarray	[3]
	MM1621	Up-regulated during growth on methanol versus acetate	Microarray	[3]
Bacteria				
Agrobacterium tumefaciens	*Atu3181*	Down-regulated by pH 5.5 versus pH 7.0	Microarray	[4]
		Down-regulated by the plant signals GABA, SA, IAA	Microarray	[5]
	Atu3633	Down-regulated by the plant signals GABA, IAA	Microarray	[5]
Bacillus subtilis	*yciC*	Zinc-dependent repression mediated by Zur	LacZ-promoter fusion/ β-Galactosidase assay; EMSA	[6]
		Up-regulated in cells grown with sulfate as sole carbon source	Microarray; LacZ fusion/ β-Galactosidase assay	[7]
Brucella suis 1330	*BR1307*	Down-regulated within murine machrophage at 48h post infection	2D-DIGE	[8]
Corynebacterium diphtheriae	*DIP1486*	Up-regulated by low Zn and low Fe or low Mn; mediated by Zur; propose also regulated by MntR and DtxR	LacZ-promoter fusion/β-galactosidase assay; qRT-PCR	[9]
Escherichia coli	*yeiR*	Up-regulated in an organic solvent tolerant strain versus wildtype under normal growth conditions and in presence of hexane	Microarray	[10]
		Up-regulated in clinical isolates displaying decreased susceptibility to tigecycline	Microarray	[11]
		Up-regulated in the presence of furonone, a quorum-sensing disrupter	Microarray	[12]
Francisella tularensis FSC033	*FTT1000c*	Protein detected only from cells isolated from spleen of infected mouse versus broth-grown cells	2D-Gel electrophoresis followed by LC–MS/MS	[13]
Francisella novicada	*FTT1000*	Down-regulated in Δ*pmrA* mutant compared to WT	Microarray	[14]
Geobacter uraniireducens	*Gura_4357*	Up-regulated in cells grown in uranium-contaminated sediment compared with cells grown in laboratory media	Microarray	[15]
Helicobacter pylori	*HP0312*	Up-regulated by growth at pH 5 compared to pH 7	Macroarray	[16]
Mycobacterium avium subsp. paratuberculosis	*MAP1730c*	Encodes second strongest antigen consistently reactive with cattle serum from cattle infected with *M. avium*	Protein array	[17]

		subsp. *avium*, *M. bovis*, and *M. avium* subsp. *paratuberculosis*		
Mycobacterium tuberculosis	*RV0106**	Zinc-dependent repression mediated by Zur	Microarray; qRT-PCR; EMSA; LacZ-promoter fusion/ β-Galactosidase assay	[18]
Pseudomonas aeruginosa	*PA2945*	Up-regulated under anaerobic conditions (cystis fibrosis simulation)	Microarray	[19]
Shewanella oneidensis MR-1	*SO1502*	Down-regulated during chromate challenge in Δ*so2426* mutant compared to WT	Multi-dimensional HPLC-MS/MS and statistical analysis	[20]
Staphylococcus epidermidis	*SE0188*	Gene in locus encoding antigen recognized by human sera	Bacteriophage lambda expression library probed with sera	[21]
Xanthomonas campestris pv *campestris*	*XC0267*	Zur-dependent expression	Microarray	[9]
Eukaryota				
Arabidopsis thaliana	*At1g80480*	Up-regulated during heat stress	Microarray	[22]
Homo sapiens	*CBWD*	Differentially expressed in SV40LT immortalized human bronchial epithelial cell line Y-BE	Suppression subtractive hybridization	[23]
		Up-regulated in normal WI-38 fibroblasts exposed to space flight stress	Suppression subtractive hybridization	[24]
	CBWD2	Down-regulated in neuroblastoma cell line exposed to a synthetic peptide corresponding to the 106–126 neurotoxic region of the cellular human prion protein	Microarray	[25]
Rattus norvegicus	*Cbwd1*	Up-regulated in response to dopamine in astrocytes cultured from rat cerebral tissues	Suppression subtractive hybridization	[26]
Saccharomyces cerevirsiae	*YNR029c*	Up-regulated in a strain with reduced azole susceptibility compared to parent	Microarray	[27]
		Transiently up-regulated 15 min after onset of fermentation in liquid fermentation media versus non-fermentative cells	Microarray	[28]
Taeniopygia guttata	*Cbwd1*	Enhanced expression in the telencephalon of males compared to females	Microarray, qRT-PCR, *in situ* hybridization	[29]

* RV0106, COG0523-like: missing canonical GTPase motifs and CXCC motif not conserved

References

1. Li Q, Li L, Rejtar T, Karger BL, Ferry JG: **Proteome of *Methanosarcina acetivorans* Part I: an expanded view of the biology of the cell.** *J Proteome Res* 2005, **4**(1):112-128.
2. Pflüger K, Ehrenreich A, Salmon K, Gunsalus RP, Deppenmeier U, Gottschalk G, Müller V: **Identification of genes involved in salt adaptation in the archaeon *Methanosarcina mazei* Gö1 using genome-wide gene expression profiling.** *FEMS Microbiol Lett* 2007, **277**(1):79-89.
3. Hovey R, Lentes S, Ehrenreich A, Salmon K, Saba K, Gottschalk G, Gunsalus RP, Deppenmeier U: **DNA microarray analysis of *Methanosarcina mazei* Gö1 reveals adaptation to different methanogenic substrates.** *Mol Genet Genomics* 2005, **273**(3):225-239.
4. Yuan ZC, Liu P, Saenkham P, Kerr K, Nester EW: **Transcriptome profiling and functional analysis of *Agrobacterium tumefaciens* reveals a general conserved response to acidic conditions (pH 5.5) and a complex acid-mediated signaling involved in Agrobacterium-plant interactions.** *J Bacteriol* 2008, **190**(2):494-507.
5. Yuan ZC, Haudecoeur E, Faure D, Kerr KF, Nester EW: **Comparative transcriptome analysis of *Agrobacterium tumefaciens* in response to plant signal salicylic acid, indole-3-acetic acid and gamma-amino butyric acid reveals signalling cross-talk and Agrobacterium--plant co-evolution.** *Cell Microbiol* 2008, **10**(11):2339-2354.
6. Gabriel SE, Miyagi F, Gaballa A, Helmann JD: **Regulation of the *Bacillus subtilis* yciC gene and insights into the DNA-binding specificity of the zinc-sensing metalloregulator Zur.** *J Bacteriol* 2008, **190**(10):3482-3488.
7. Auger S, Danchin A, Martin-Verstraete I: **Global expression profile of *Bacillus subtilis* grown in the presence of sulfate or methionine.** *J Bacteriol* 2002, **184**(18):5179-5186.
8. Al Dahouk S, Jubier-Maurin V, Scholz H, Tomaso H, Karges W, Neubauer H, Köhler S: **Quantitative analysis of the intramacrophagic *Brucella suis* proteome reveals metabolic adaptation to late stage of cellular infection.** *Proteomics* 2008, **8**(18):3862-3870.
9. Huang DL, Tang DJ, Liao Q, Li HC, Chen Q, He YQ, Feng JX, Jiang BL, Lu GT, Chen B *et al*: **The Zur of *Xanthomonas campestris* functions as a repressor and an activator of putative zinc homeostasis genes via recognizing two distinct sequences within its target promoters.** *Nucleic Acids Res* 2008, **36**(13):4295-4309.
10. Hayashi S, Aono R, Hanai T, Mori H, Kobayashi T, Honda H: **Analysis of organic solvent tolerance in *Escherichia coli* using gene expression profiles from DNA microarrays.** *J Biosci Bioeng* 2003, **95**(4):379-383.

11. Keeney D, Ruzin A, McAleese F, Murphy E, Bradford PA: **MarA-mediated overexpression of the AcrAB efflux pump results in decreased susceptibility to tigecycline in *Escherichia coli*.** *J Antimicrob Chemother* 2008, **61**(1):46-53.
12. Ren D, Bedzyk LA, Ye RW, Thomas SM, Wood TK: **Differential gene expression shows natural brominated furanones interfere with the autoinducer-2 bacterial signaling system of *Escherichia coli*.** *Biotechnol Bioeng* 2004, **88**(5):630-642.
13. Twine SM, Mykytczuk NC, Petit MD, Shen H, Sjöstedt A, Wayne Conlan J, Kelly JF: ***In vivo* proteomic analysis of the intracellular bacterial pathogen, *Francisella tularensis*, isolated from mouse spleen.** *Biochem Biophys Res Commun* 2006, **345**(4):1621-1633.
14. Mohapatra NP, Soni S, Bell BL, Warren R, Ernst RK, Muszynski A, Carlson RW, Gunn JS: **Identification of an orphan response regulator required for the virulence of *Francisella spp*. and transcription of pathogenicity island genes.** *Infect Immun* 2007, **75**(7):3305-3314.
15. Holmes DE, O'Neil RA, Chavan MA, N'Guessan LA, Vrionis HA, Perpetua LA, Larrahondo MJ, DiDonato R, Liu A, Lovley DR: **Transcriptome of *Geobacter uraniireducens* growing in uranium-contaminated subsurface sediments.** *ISME J* 2009, **3**(2):216-230.
16. Bury-Moné S, Thiberge JM, Contreras M, Maitournam A, Labigne A, De Reuse H: **Responsiveness to acidity via metal ion regulators mediates virulence in the gastric pathogen *Helicobacter pylori*.** *Mol Microbiol* 2004, **53**(2):623-638.
17. Bannantine JP, Paustian ML, Waters WR, Stabel JR, Palmer MV, Li L, Kapur V: **Profiling bovine antibody responses to *Mycobacterium avium* subsp. *paratuberculosis* infection by using protein arrays.** *Infect Immun* 2008, **76**(2):739-749.
18. Maciag A, Dainese E, Rodriguez GM, Milano A, Provvedi R, Pasca MR, Smith I, Palù G, Riccardi G, Manganelli R: **Global analysis of the *Mycobacterium tuberculosis* Zur (FurB) regulon.** *J Bacteriol* 2007, **189**(3):730-740.
19. Platt MD, Schurr MJ, Sauer K, Vazquez G, Kukavica-Ibrulj I, Potvin E, Levesque RC, Fedynak A, Brinkman FS, Schurr J *et al*: **Proteomic, microarray, and signature-tagged mutagenesis analyses of anaerobic *Pseudomonas aeruginosa* at pH 6.5, likely representing chronic, late-stage cystic fibrosis airway conditions.** *J Bacteriol* 2008, **190**(8):2739-2758.
20. Chourey K, Thompson MR, Shah M, Zhang B, Verberkmoes NC, Thompson DK, Hettich RL: **Comparative temporal proteomics of a response regulator (SO2426)-deficient strain and wild-type *Shewanella oneidensis* MR-1 during chromate transformation.** *J Proteome Res* 2009, **8**(1):59-71.

21. Pourmand MR, Clarke SR, Schuman RF, Mond JJ, Foster SJ: **Identification of antigenic components of *Staphylococcus epidermidis* expressed during human infection.** *Infect Immun* 2006, **74**(8):4644-4654.
22. Steinhauser D, Usadel B, Luedemann A, Thimm O, Kopka J: **CSB.DB: a comprehensive systems-biology database.** *Bioinformatics* 2004, **20**(18):3647-3651.
23. An Q, Pacyna-Gengelbach M, Schlüns K, Deutschmann N, Guo S, Gao Y, Zhang J, Cheng S, Petersen I: **Identification of differentially expressed genes in immortalized human bronchial epithelial cell line as a model for in vitro study of lung carcinogenesis.** *Int J Cancer* 2003, **103**(2):194-204.
24. Liu Y, Wang E: **Transcriptional analysis of normal human fibroblast responses to microgravity stress.** *Genomics Proteomics Bioinformatics* 2008, **6**(1):29-41.
25. Martínez T, Pascual A: **Identification of genes differentially expressed in SH-SY5Y neuroblastoma cells exposed to the prion peptide 106-126.** *Eur J Neurosci* 2007, **26**(1):51-59.
26. Shi J, Cai W, Chen X, Ying K, Zhang K, Xie Y: **Identification of dopamine responsive mRNAs in glial cells by suppression subtractive hybridization.** *Brain Res* 2001, **910**(1-2):29-37.
27. Barker KS, Pearson MM, Rogers PD: **Identification of genes differentially expressed in association with reduced azole susceptibility in .** *J Antimicrob Chemother* 2003, **51**(5):1131-1140.
28. Tanaka F, Ando A, Nakamura T, Takagi H, Shima J: **Functional genomic analysis of commercial baker's yeast during initial stages of model dough-fermentation.** *Food Microbiol* 2006, **23**(8):717-728.
29. Wade J, Tang YP, Peabody C, Tempelman RJ: **Enhanced gene expression in the forebrain of hatchling and juvenile male zebra finches.** *J Neurobiol* 2005, **64**(2):224-238.
