# Supplementary figures and images for: A subset of the diverse COG0523 family of putative metal chaperones is linked to zinc homeostasis in all kingdoms of life
Source: BMC Genomics. 2009 Oct 12;10:470. doi: 10.1186/1471-2164-10-470 (PMC2770081; doi:10.1186/1471-2164-10-470)

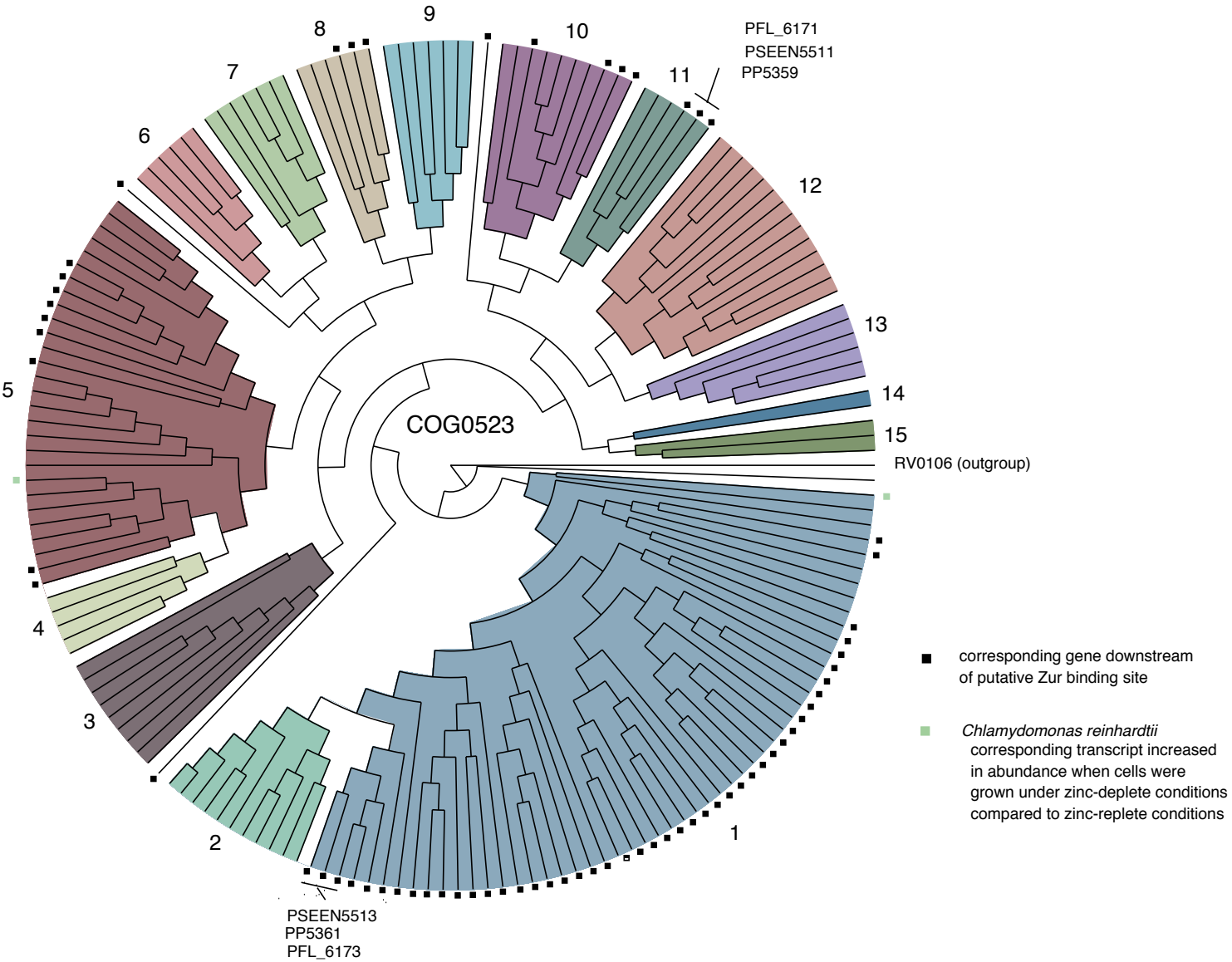

Supplement: Additional file 2 — Phylogeny of COG0523 subgroups. A, Each identified subgroup is shaded and labeled. The branches representing proteins encoded by putative Zur-regulated genes are marked with a black square. The branches representing C. reinhardtii COG0523 homologs encoded by the genes induced by zinc deficiency are marked with a green square. Branches representing the Pseudomonas paralogs discussed in the text are labeled. Protein IDs for each branch can be found in Additional File 7. [file 1471-2164-10-470-S2.PDF]
