# Supplementary material for: A subset of the diverse COG0523 family of putative metal chaperones is linked to zinc homeostasis in all kingdoms of life
Source: BMC Genomics. 2009 Oct 12;10:470. doi: 10.1186/1471-2164-10-470 (PMC2770081; doi:10.1186/1471-2164-10-470)
Supplement: Additional file 3 — Detailed description of each COG0523 subfamily. For subfamilies 2-15, representative genomic context figures are given as is a list of the locus-tags for members of each subfamily identified by physical clustering to common genes. [file 1471-2164-10-470-S3.PDF]

**Additional File 3.**
**Detailed description of the identified COG0523 subgroups.**

**Subgroup 1** Described in main body of text.

**Subgroup 2**

| Genome | Locus-tag | Genbank accession no. | Ref |
|---|---|---|---|
| *Microbacterium* sp. AJ115 | nha3 | CAG29801.1 | [1] |
| *Pseudomonas chlororaphis* B23 | P47K | P31521.1 | [2] |
| *Pseudomonas* sp. K-9 | nhr | BAD98534.1 | [3] |
| *Rhodococcus erythropolis* A4 | nhr3 | CAQ16890.1 | [4] |
| *Rhodococcus* sp. N-771 | nha3 | BAA36599.1 | [5, 6] |
| *Rhodococcus* sp. N-774 | ORF1188 | BAA06274.1 | [7] |
| *Rhodococcus globerulus* A-4 | nhr3 | BAC99082.1 | [8] |
| *Rhodococcus jostii* RHA1 | RHA1_ro00362 | ABG92198.1 | |
| *Acinetobacter baylyi* ADP1 | ACIAD1614 | YP_046285.1 | |
| *Pseudomonas putida* F1 | Pput_2730 | ABQ78864.1 | |
| *Burkholderia ambifaria* AMMD | Bamb_6542 | YP_778420.1 | |
| *Burkholderia ambifaria* MC40-6 | BamMC406_6256 | ACB68689.1 | |
| *Burkholderia cenocepacia* AU 1054 | Bcen_4084 | YP_623946.1 | |
| *Burkholderia cenocepacia* MC0-3 | Bcenmc03_3235 | YP_001776881.1 | |

**Table S1: Fe-type nitrile hydratase activator proteins.** Identity based on sequence homology and genome context.

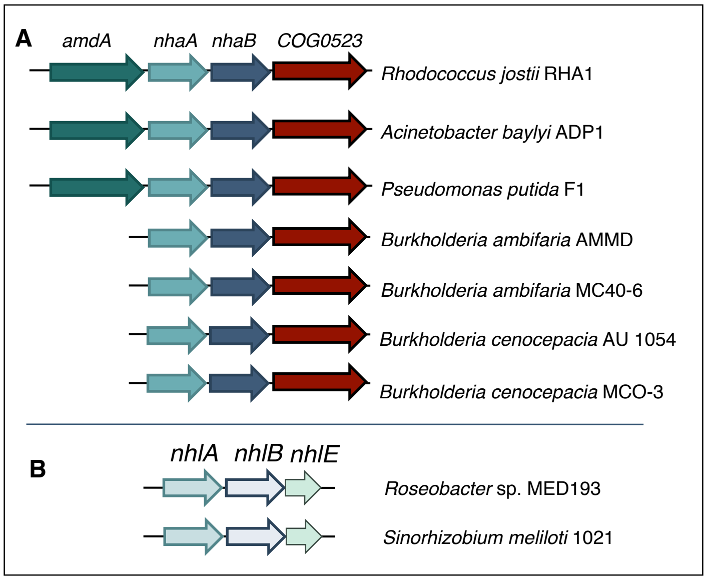


**Figure S1: Genome context of predicted nitrile hydratase activators.** (A) Gene clusters of predicted Fe-type nitrile hydratase subunits and corresponding activator proteins. A paralog of COG0523, the activator, is found upstream of the α- and β- subunits of the Fe-type NHase. NhaA contains the $Fe^{3+}$-binding motif, CSLCSCT. (B) Gene cluster of representative, predicted Co-type nitrile hydratase activator subunits. NhlE, which shares no sequence homology with COG0523, is found upstream of the Co-type nitrile hydratase subunits. NhlA contains the $Co^{2+}$-binding motif, CTLCSCY.

## Subgroup 3

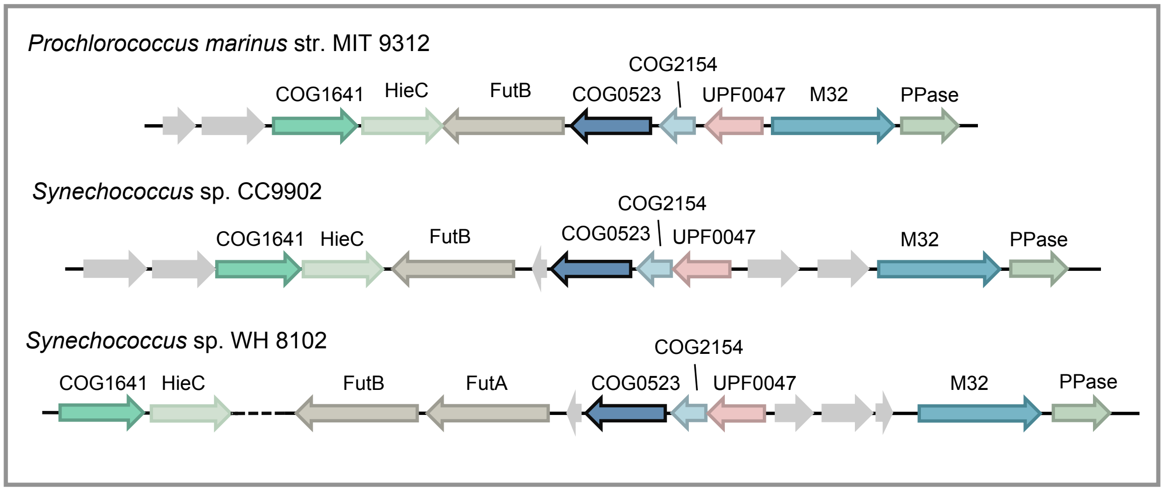


**Figure S2: Genome context of subgroup 3 members.**

Subgroup 3 co-localizes on the genome with homologs of uncharacterized conserved proteins (COG1641), putative transmembrane protein (HieC), Ferric iron ABC transporter, permease protein (FutB) and periplasmic binding protein (FutA), Pterin-4-alpha-carbinolamine dehydratase (COG2154), protein of unknown function (UPF0047), carboxypeptidase Taq (M32) metallopeptidase (M32), and inorganic pyrophosphatase (PPase).

Characterized members of COG2154 from animals have been shown to be responsible for recycling pterin cofactors generated by aromatic amino acid hydroxylases (AAHs) (for a review see [9]). In bacteria, the absence of AAH in many COG2154-encoding genomes suggests its role in the recycling of pterin cofactors from other pterin-dependent enzymes[10]. UPF0047 appears to be a metalloenzyme; the crystal structure of UPF0047 from *Sulfolobus tokodaii* has been solved and found to co-crystalize with a zinc ion in a potential catalytic site [11]. Over-expression of the UPF0047 homolog from *Escherichia coli* as well as *Thermotoga, Sulfolobus,* and *Pyrococcus* was found to complement the thiamine auxotrophy of a Δ*thiE E. coli* mutant [12].

| **Genome** | **Locus_tag** |
|---|---|
| *Synechococcus* sp. CC9311 | Sync_2045 |
| *Synechococcus* sp. CC9605 | Syncc9605_0672 |
| *Synechococcus* sp. RS9917 | RS9917_09326 |
| *Synechococcus* sp.WH 7805 | WH7805_13878 |
| *Synechococcus* sp. WH 8102 | SYNW1795 |
| *Prochlorococcus marinus* (5 genomes) | PMT9312_0491 (str. MIT 9312) |

**Table S2. Genomes where subgroup 3 gene clusters occur.**

## Subgroup 4

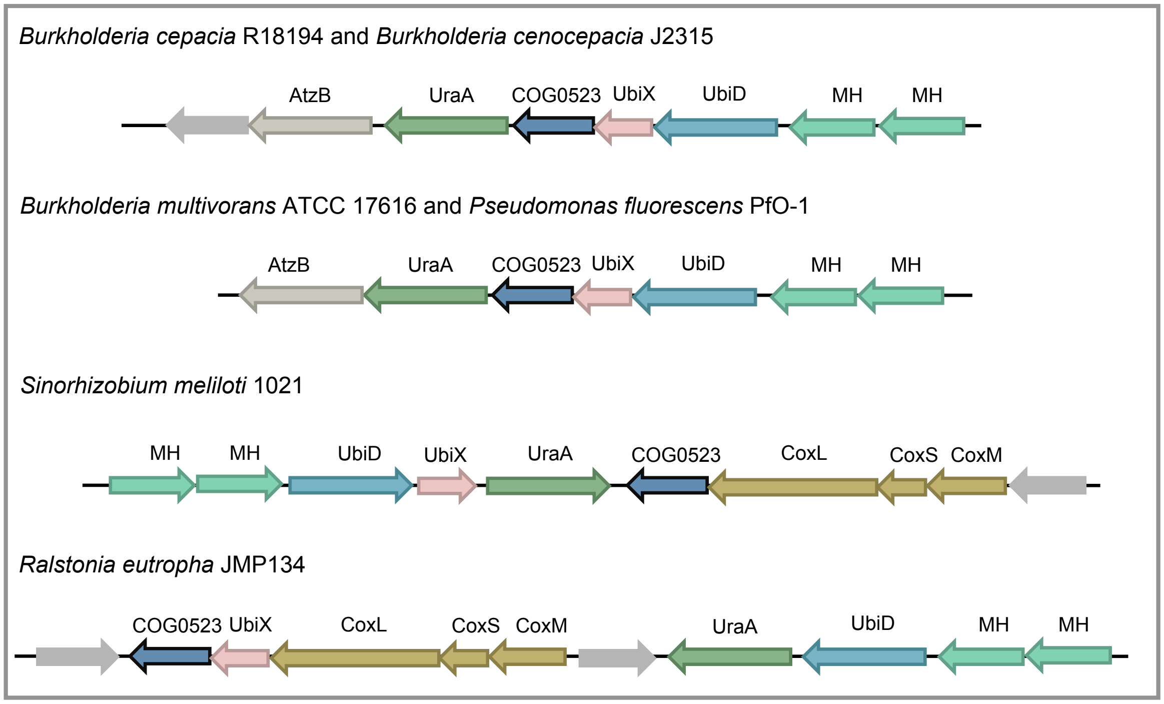


**Figure S3: Genome context of subgroup 4 members.**

Subgroup 4 co-localizes on the genome with genes encoding metal-dependent hydrolases (MH), xanthine/uracil permease (UraA), 3-polyprenyl-4-hydroxybenzoate carboxy-lyase (UbiX and UbiD), hydroxyatrazine ethylaminohydrolase (AtzB), molybdenum-containing hydroxylase (CoxLSM). Several of these genes encode various proteins involved in the metabolism and degradation of aromatic compounds. In addition to molybdenum, Mo-containing hydroxylases have been found to contain [2Fe-2S] clusters (For a review see [13]).

| Organism | Locus_tag |
|---|---|
| *Sinorhizobium meliloti* 1021 | SMb20133 |
| *Burkholderia cenocepacia* J2315 | BCAM2270 |
| *Burkholderia cepacia* R18194 | Bcep18194_B0634 |
| *Ralstonia eutropha* JMP134 | Reut_A3078 |
| *Pseudomonas fluorescens* PfO-1 | Pfl_3432 |

**Table S3. Genomes where subgroup 4 gene clusters occur.**

## Subgroup 5

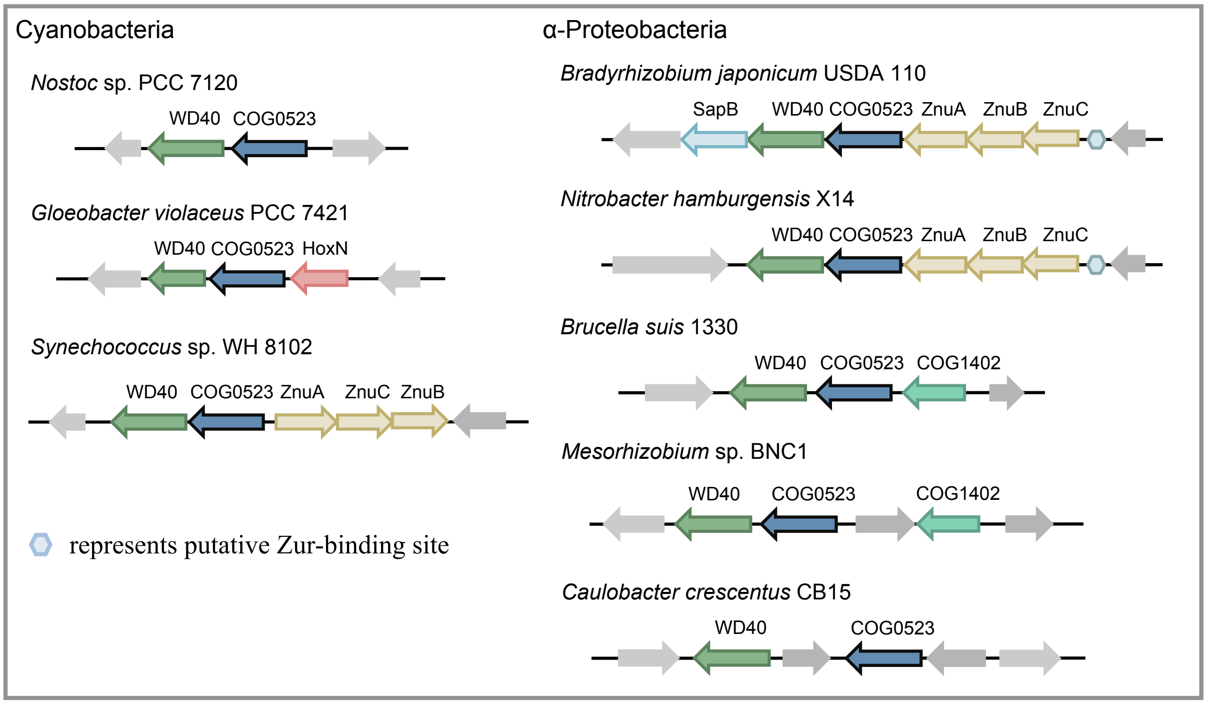


**Figure S4: Genome context of subgroup 5 members.**

Subgroup 5 co-localizes on the genome with genes encoding metal transporters (HoxN/HupN/NixA family cobalt transporter (HoxN) and high-affinity zinc transporter (ZnuABC)), WD40 repeat-containing protein (WD40), uncharacterized membrane family (SapB), and creatine amidohydrolase-like (COG1402).

| Genome | Locus_tag |
|---|---|
| *Synechococcus* sp. WH 8102 | SYNW2482 |
| *Gloeobacter violaceus* PCC 7421 | glr0534 |
| *Anabaena variabilis* ATCC 29413 | Ava_0285 |
| *Nostoc punctiforme* PCC 73102 | Npun_R3841 |
| *Nostoc* sp. PCC 7120 | all1751 |
| *Prochlorococcus marinus* (5 genomes) | P9211_15201 (str. MIT 9211) |
| *Caulobacter* sp. K31 | Caul_0196 |
| *Caulobacter crescentus* CB15 | CC0321 |
| *Aurantimonas* sp. SI85-9A1 | SI859A1_00649 |
| *Bartonella henselae* str. Houston-1 | BH12980 |
| *Bradyrhizobium japonicum* USDA 110 | bll7768 |
| *Nitrobacter hamburgensis* X14 | Nham_3425 |
| *Nitrobacter winogradskyi* Nb-255 | Nwi_0917 |
| *Rhodopseudomonas palustris* (4 genomes) | RPE_4794 (str. BisB53) |
| *Brucella abortus* biovar 1 str. 9-941 | BruAb2_0247 |

| | |
|---|---|
| *Brucella canis* ATCC 23365 | BCAN_B1006 |
| *Brucella melitensis* 16M | BMEII0308 |
| *Brucella suis* 1330 | BRA0987 |
| *Brucela suis* ATCC 23445 | BSUIS_B0982 |
| *Mesorhizobium loti* MAFF303099 | mll5156 |
| *Mesorhizobium* sp. BNC1 | MBNC02001409 |
| *Rhizobium leguminosarum* bv. *viciae* 3841 | RL4148 |
| *Sinorhizobium meliloti* 1021 | SMc02978 |
| *Agrobacterium tumefaciens* str. C58 | Atu4502 |
| *Acidiphilium cryptum* JF-5 | Acry_1304 |
| *Gluconobacter oxydans* 621H | GOX1617 |
| *Granulibacter bethesdensis* CGDNIH1 | GbCGDNIH1_0170 |
| *Bordetella bronchiseptica* RB50 | BB0682 |
| *Bordetella parapertussis* 12822 | BPP0675 |

**Table S4. Genomes where subgroup 5 gene clusters occur.**

## Subgroup 6

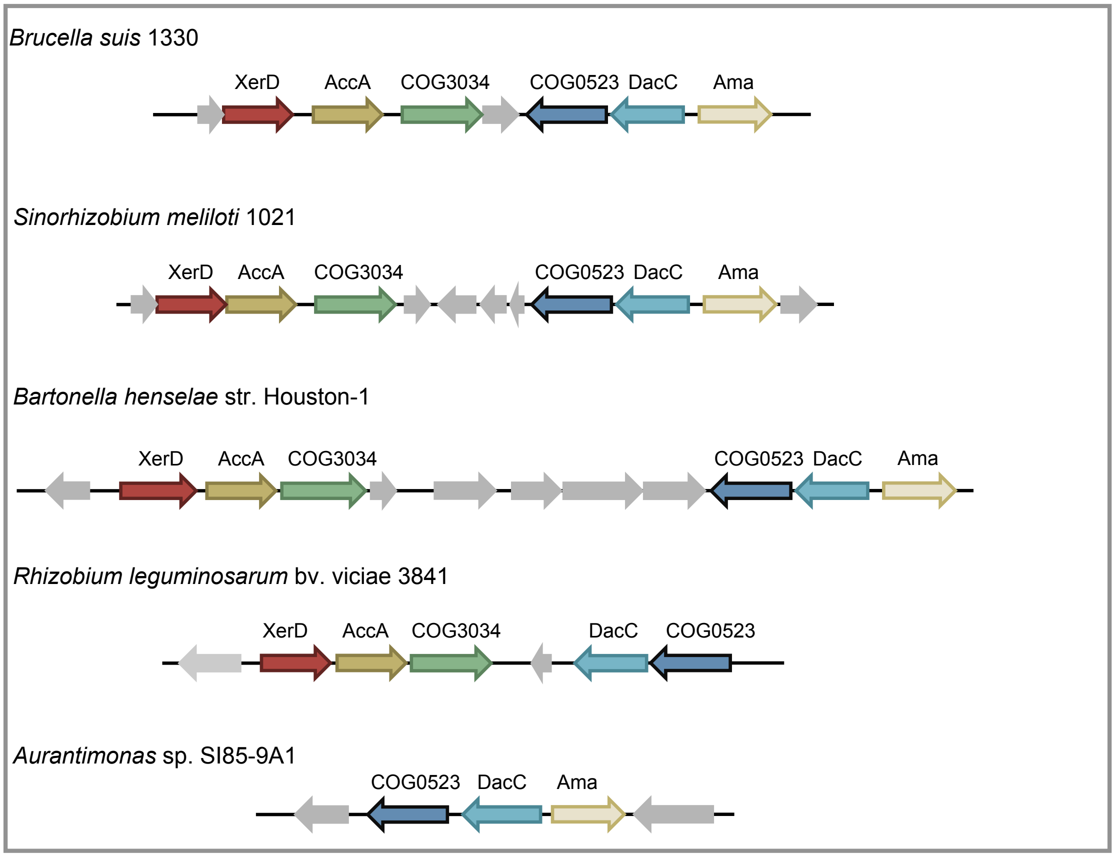


**Figure S5: Genome context of subgroup 6 members.**

Subgroup 6 co-localizes with genes encoding integrase/recombinase (XerD), acetyl-coenzyme A carboxyl transferase alpha chain (AccA), uncharacterized protein (COG3034), D-alanyl-D-alanine carboxypeptidase (DacC), and N-acyl-L-amino acid amidohydrolase (Ama).

| **Organism** | **Locus_tag** |
|---|---|
| *Parvularcula bermudensis* HTCC2503 | PB2503_00115 |
| *Aurantimonas* sp. SI85-9A1 | SI859A1_02956 |
| *Bartonella henselae* str. Houston-1 | BH16310 |
| *Brucella abortus* biovar 1 str. 9-941 | BruAb1_2010 |
| *Brucella canis* ATCC 23365 | BCAN_A2081 |
| *Brucella melitensis* 16M | BMEI0036 |
| *Brucella suis* (2 genomes) | BR2035 (str. 1330) |
| *Methylobacterium extorquens* PA1 | Mext_1219 |
| *Mesorhizobium loti* MAFF303099 | mll3580 |
| *Mesorhizobium* sp. BNC1 | MBNC02000233 |
| *Agrobacterium tumefaciens* str. C58 | Atu3633 |
| *Rhizobium leguminosarum* bv. *viciae* 3841 | RL4362 |
| *Sinorhizobium meliloti* 1021 | SMc00684 |

**Table S5. Genomes containing subgroup 6 gene clusters.**

## Subgroup 7

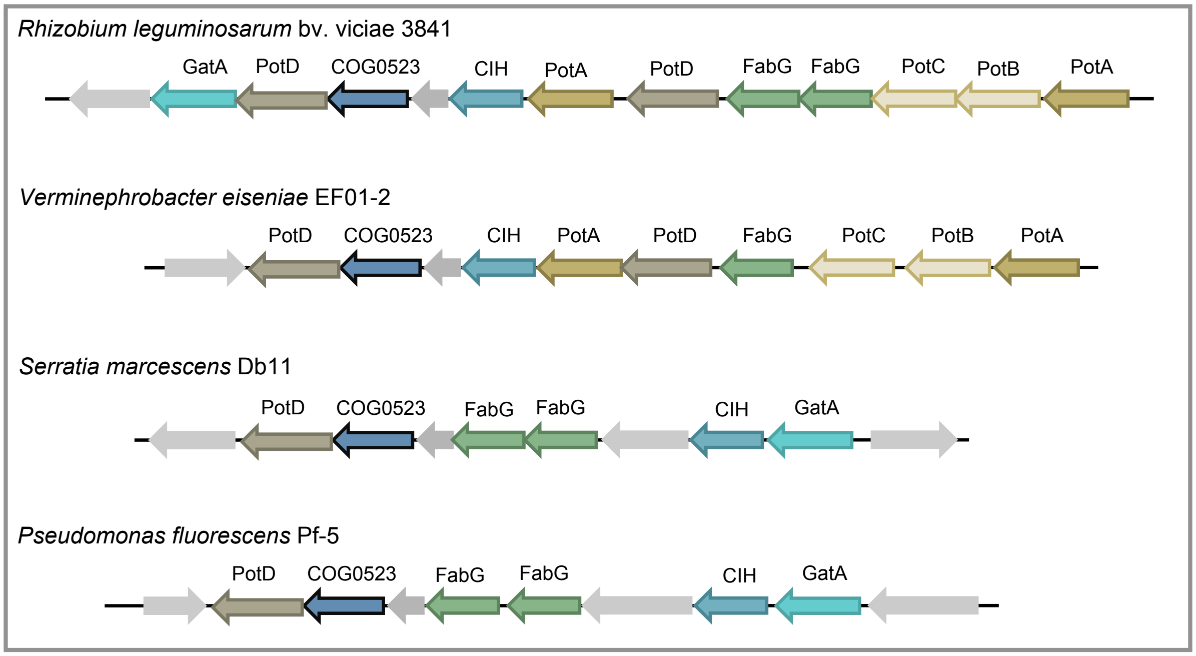


**Figure S6: Genome context of subgroup 7 members.**

Subgroup 7 co-localizes with genes encoding Asp-tRNAAsn/Gu-tRNAGln amidotransferase A subunit and related amidases family protein (GatA), spermine/putrescine periplasmic binding protein (PotD), spermine/putrescine import ATP-binding protein (PotA), spermine/putrescine transport permease protein (PotB and PotC), cyclic imide hydrolase (CIH), and 3-ketoacyl-(acyl-carrier-protein) reductase (FabG).

| Organism | Locus_tag |
|---|---|
| *Rhizobium leguminosarum* bv. viciae 3841 | pRL120796 |
| *Verminephrobacter eiseniae* EF01-2 | Veis_1001 |
| *Serratia marcescens* DB11 | figl615.1.peg.4294 |
| *Pseudomonas fluorescens* Pf-5 | PFL_1367 |
| *Pseudomonas syringae* (3 genomes) | PSPTO4198 (pv. tomato str. DC3000) |

**Table S6. Genomes containing subgroup 7 gene clusters.**

**Subgroup 8**

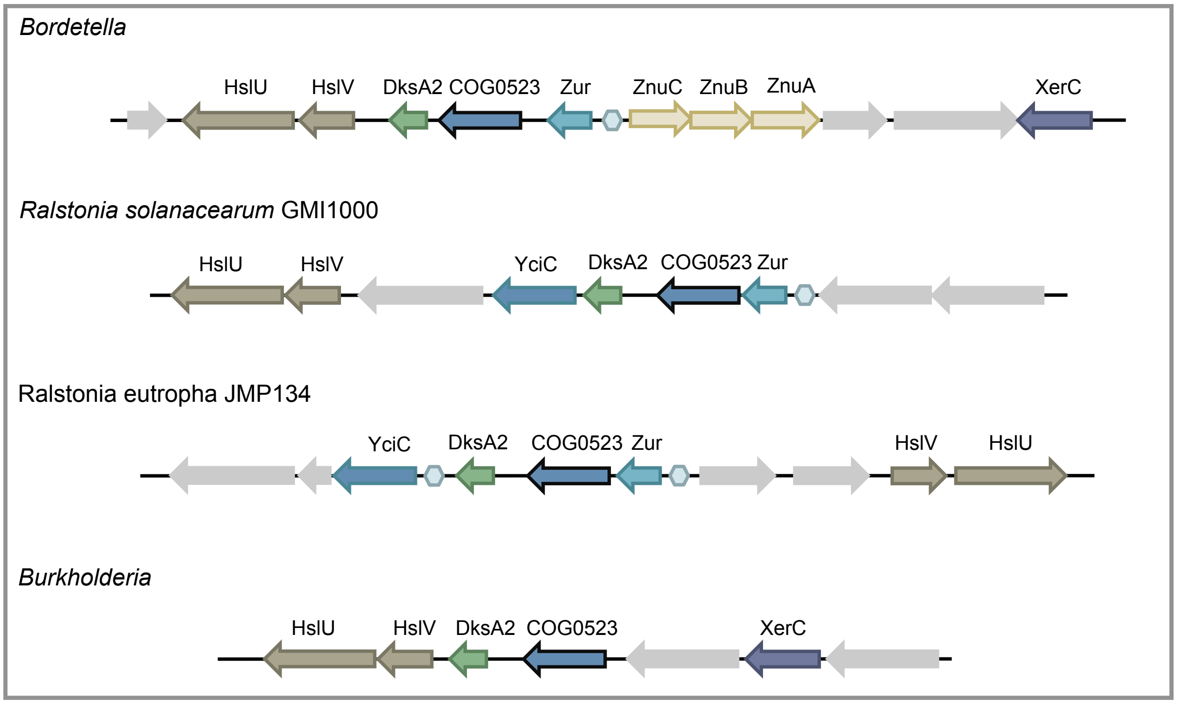


**Figure S7: Genome context of subgroup 8 members.**

Subgroup 8 clusters with ATP-dependent hsl protease ATP-binding subunit (HslU), ATP-dependent protease (HslV), C4-type zinc finger DksA/TraR family protein (DksA2), zinc uptake regulation protein (Zur), high affinity zinc transporter (ZnuABC), integrase/recombinase (XerC), and the COG0523 Subgroup 2 paralog (YciC).

| Organism | Locus_tag |
|---|---|
| *Bordetella avium* | BAV0145 |
| *Bordetella bronchiseptica* RB50 | BB0181 |
| *Bordetella parapertussis* 12822 | BPP0179 |
| *Bordetella pertussis* Tohoma I | BP3084 |
| *Burkholderia ambifaria* AMMD | Bamb_3135 |
| *Burkholderia cenocepacia* (4 genomes) | Bcen_2475 (str. AU 1054) |
| *Burkholderia cepacia* R18194 | Bcep18194_A6439 |
| *Burkholderia dolosa* AUO158 | BDAG_00307 |
| *Burkholderia multivorans* ATCC 17616 | Bmul_3084 |
| *Burkholderia cepacia* R1808 | Bucepa02001131 |
| *Burkholderia vietnamiensis* strain G4 | Bcep1808_3172 |
| *Burkholderia fungorum* | Bcep2993 |
| *Burkholderia mallei* (3 genomes) | BMA10229_A2171 (str. 10229) |
| *Burkholderia xenovorans* LB400 | Bxe_A4377 |
| *Burkholderia pseudomallei* (4 genomes) | BURPS1655_K0002 (str. 1655) |
| *Cupriavidus metallidurans* CH34 | Rmet_0127 |
| *Ralstonia eutropha* JMP134 | Reut_A0163 |
| *Polynucleobacter* sp. QLW-P1DMWA-1 | Pnuc_2015 |
| *Ralstonia solanacearum* GMI1000 | RSc0047 |
| *Acidovorax avenae* subsp. citrulli AAC00-1 | Aave_0807 |
| *Acidovorax* sp. JS42 | Ajs_3684 |
| *Delftia acidovorans* SPH-1 | Daci_1456 |
| *Polaromonas* sp. JS666 | Bpro_1063 |
| *Rhodoferax ferrireducens* DSM 15236 | Rfer_3439 |
| *Verminephrobacter eiseniae* EF01-2 | Veis_4556 |
| *Herminiimonas arsenicoxydans* | HEAR2959 |
| *Leptothrix cholodni* SP-6 | Lcho_3834 |

**Table S7. Genomes containing Subgroup 8 gene clusters.**

**Subgroup 9**

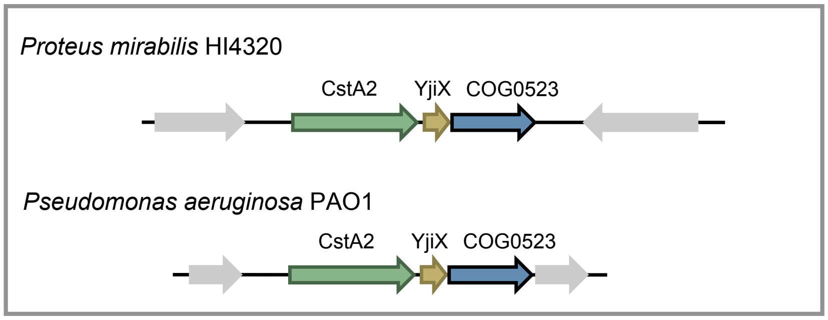


**Figure S8: Genome context of subgroup 9 members.**

Subgroup 9 co-localizes with genes encoding a carbon starvation protein paralog (CstA2) and a protein of unknown function (YjiX).

| Genome | Locus_tag |
|---|---|
| *Enterobacter* sp. 638 | Ent638_0509 |
| *Escherichia coli* (17 genomes) | YjiA |
| *Klebsiella pneumoniae* MGH 78578 | KPN_04774 |
| *Erwinia carotovora* subsp. *atroseptica* SCRI1043 | ECA1189 |
| *Proteus mirabilis* HI4320 | PMI0144 |
| *Salmonella bongori* 12149 | figl12149.1.peg.4488 |
| *Salmonella enterica* (5 genomes) | STY4888 (subsp. Enterica serovar Typhi str. CT18) |
| *Salmonella typhimurium* LT2 | STM4530 |
| *Serratia marcescens* Db11 | figl615.1.peg.4294 |
| *Serratia proteamaculans* 568 | Spro_0580 |
| *Shigella dysenteriae* Sd197 | SDY_4605 |
| *Shigella flexneri* 2a (2 genomes) | S4640 (str. 2457T) |
| *Shigella sonnei* (2 genomes) | SSO_4485 (str. Ss046) |
| *Azotobacter vinelandii* | Avin3722 |
| *Pseudomonas aeruginosa* | PA4604 (str. PAO1) |
| *Pseudomonas entomophila* L48 | PSEEN0805 |
| *Pseudomonas fluorescens* (3 genomes) | PFL_5350 (str. Pf-5) |
| *Pseudomonas putida* (4 genomes) | Pput_4501 (str. F1) |
| *Pseudomonas syringae* (3 genomes) | Psyr_4271 (pv. Syringae B728a) |

**Table S8. Genomes containing Subgroup 9 gene clusters.**

**Subfamily 10**

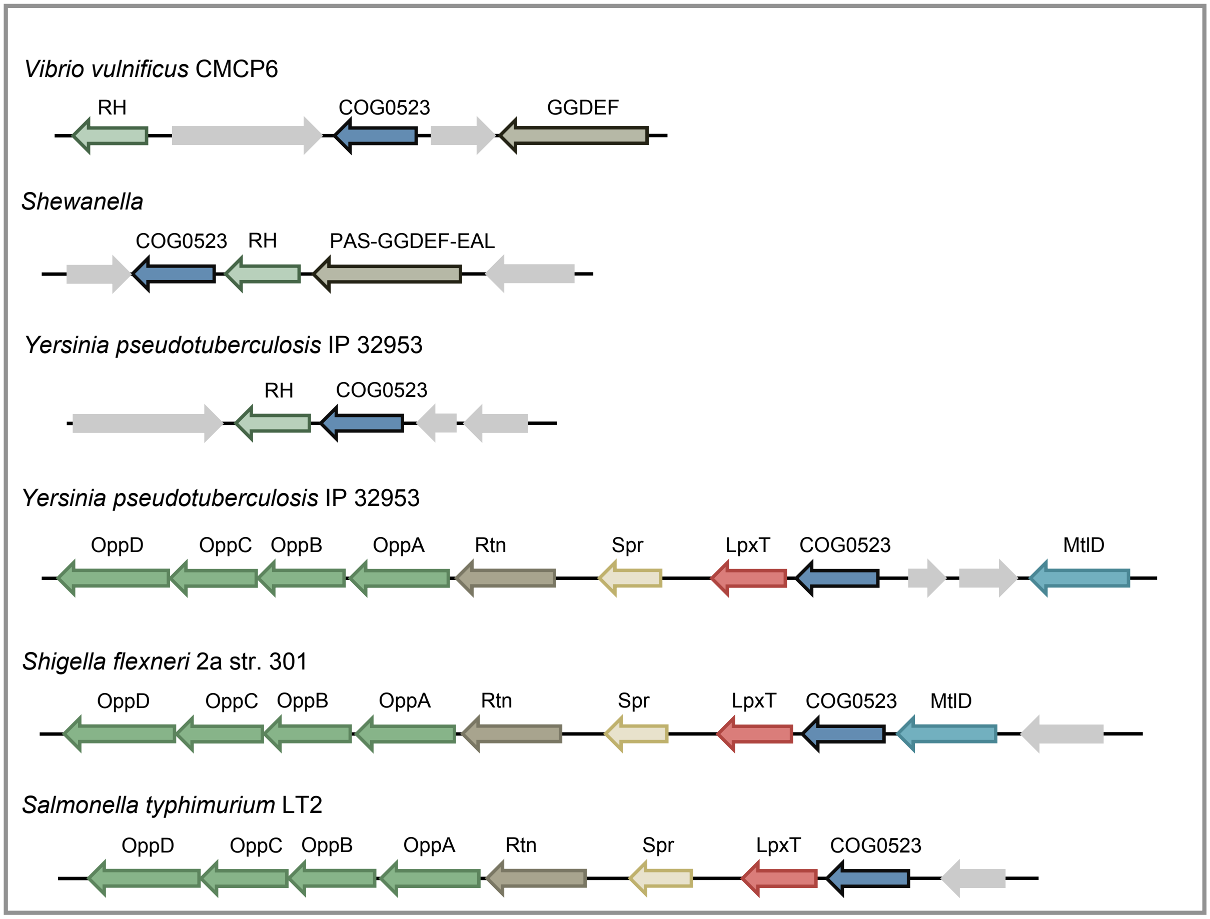


**Figure S9: Genome context of subgroup 10 members.**

Subfamily 10 co-localizes with genes encoding signal transduction proteins (GGDEF, PAS-GGDEF-EAL, or Rtn) or an ATP-dependent RNA helicase (RH). Abbreviations: MtlD, mannitol-1-phosphate/altronate dehydrogenases; LpxT, undecaprenyl pyrophosphate phosphatase; Spr, predicted peptidase; OppABCD, putative ATP-dependent oligopeptide permease.

| Genome | Locus_tag |
|---|---|
| *Enterobacter* sp. 638 | Ent638_2769 |
| *Escherichia coli* (17 genomes) | YeiR |
| *Klebsiella pneumoniae* MGH 78578 | KPN_02606 |
| *Erwinia carotovora* subsp. *atroseptica* SCRI1043 | ECA2733 |
| *Salmonella bongori* 12149 | figl12149.1.peg.2276 |
| *Salmonella enterica* (5 genomes) | STY2448 (subsp. *enterica* serovar *Typhi* str. CT18) |

| | |
|---|---|
| *Salmonella typhimurium* LT2 | STM2212 |
| *Serratia marcescens* Db11 | figl615.1.peg.3296 |
| *Serratia proteamaculans* 568 | Spro_3241 |
| *Shigella dysenteriae* Sd197 | SDY_0906 |
| *Shigella flexneri 2a* (2 genomes) | SF2260 (str. 301) |
| *Shigella sonnei* (2 genomes) | SSO_2229 (str. Ss046) |
| *Yersinia bercovieri* ATCC 43970 | YberA_01001247 |
| *Yersinia enterocolitica* 8081 | YE1435 |
| *Yersinia frederiksenii* ATCC 33641 | YfreA_01001244 |
| *Yersinia intermedia* ATCC 29909 | YintA_01003502 |
| *Yersinia mollaretii* ATCC 43969 | YmolA_01001161 |
| *Yersinia pestis* (7 genomes) | YPA_0995 (str. Antiqua) |
| *Yersinia pseudotuberculosis* (3 genomes) | YPTB1311 (str. IP 32953) |
| *Shewanella amozonensis* SB2B | Sama_2446 |
| *Shewanella baltica* (4 genomes) | Sbal_1338 (str. OS155) |
| *Shewanella denitrificans* OS217 | Sden_2575 |
| *Shewanella frigidimarina* NCIMB 400 | Sfri_2795 |
| *Shewanella halifaxensis* HAW-EB4 | Shal_3090 |
| *Shewanella* sp. PV-4 | Shew_2776 |
| *Shewanella oneidensis* MR-1 | SO1502 |
| *Shewanella pealeana* ATCC 700345 | Spea_3001 |
| *Shewanella putrefaciens* CN-32 | Sputcn32_1255 |
| *Shewanella sediminis* HAW-EB3 | Ssed_3334 |
| *Shewanella* sp. MR-4 | Shewmr4_2751 |
| *Shewanella* sp. MR-7 | Shewmr7_2829 |
| *Shewanella* sp. W3-18-1 | Sputw3181_2849 |
| *Shewanella* sp. ANA-3 | Shewana3_2927 |
| *Vibrio alginolyticus* 12G01 | V12G01_06973 |
| *Vibrio parahaemolyticus* RIMD 2210633 | VPA0589 |
| *Vibrio* sp. Ex25 | VEx2w_01001184 |
| *Vibrio* sp. MED222 | MED222_06985 |
| *Vibrio splendidus* 12B01 | V12B01_03988 |
| *Vibrio vulnificus* (2 genomes) | VV20385 (str. CMCP6) |

**Table S9. Genomes containing Subgroup 10 gene clusters.**

## Subgroup 11

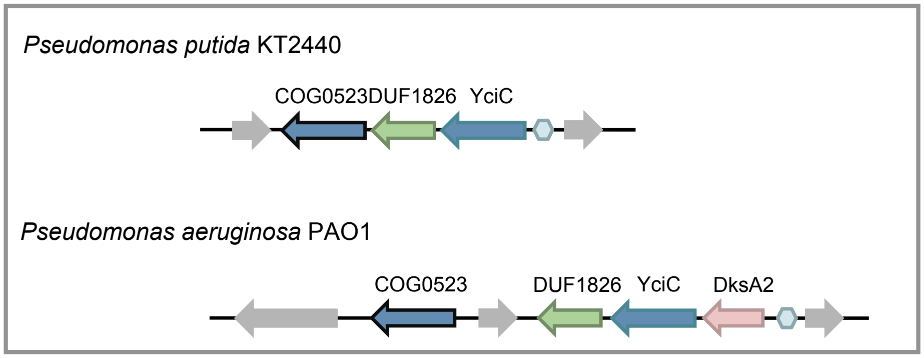


**Figure S10: Genome context of subgroup 11 members.**

Subgroup 11 co-localizes with genes encoding an uncharacterized protein (DUF1826), COG0523 homolog from subfamily 2 (YciC), and C4-type zinc finger DksA/TraR family protein (DksA2).

| Genome | Locus_tag |
|---|---|
| *Pseudomonas aeruginosa* (3 genomes) | PA5532 (str. PAO1) |
| *Pseudomonas mendocina* ymp | Pmen_4533 |
| Pseudomonas entomophila L48 | PSEEN5511 |
| *Pseudomonas fluorescens* (3 genomes) | PFL_6171 (str. Pf-5) |
| *Pseudomonas putida* (4 genomes) | Pput_5267 (str. F1) |
| *Pseudomonas syringae* (3 genomes) | Psyr_5072 (pv. Syringae B278a) |

**Table S10. Genomes containing Subgroup 11 gene clusters.**

## Subgroup 12

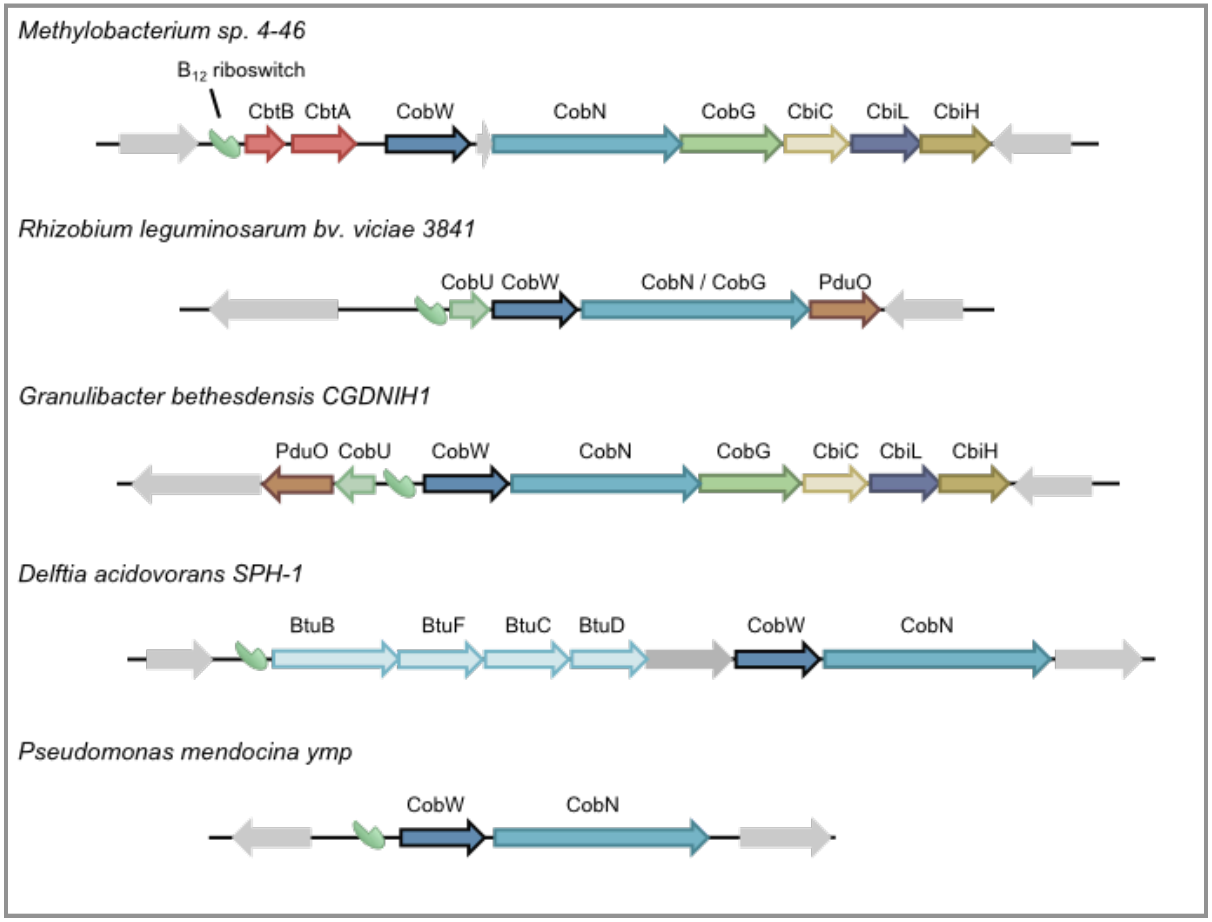


**Figure S11: Genome context of subgroup 12 members.**

Abbreviations: CobW, subfamily 12 COG0523 paralog; CbtB, Cobalt transporter subunit; CbtA, cobalt transporter subunit; CobN, cobaltochelatase subunit; CobG, Precorrin-3B synthase; CbiC, cobalt-precorrin-8x methylmutase; CbiL, Cobalt-precorrin-2 C20-methyltransferase; CbiL, Cobalt-precorrin-3b C17-methyltransferase; CbiH, Cobalt-precorrin-6x reductase; CobU, Adenosylcobinamide-phosphate guanylyltransferase; PduO, Cob(I)alamin adenosyltransferase; BtuB, Outer membrane vitamin B12 receptor; BtuF, Vitamin B12 ABC transporter, B12-binding component; BtuC, Vitamin B12 ABC transporter, permease component; BtuD, Vitamin B12 ABC transporter, ATPase component.

Genomes encoding CobW that are downstream of a putative $B_{12}$ riboswitch are listed in Additional file 4.

## Subgroup 13

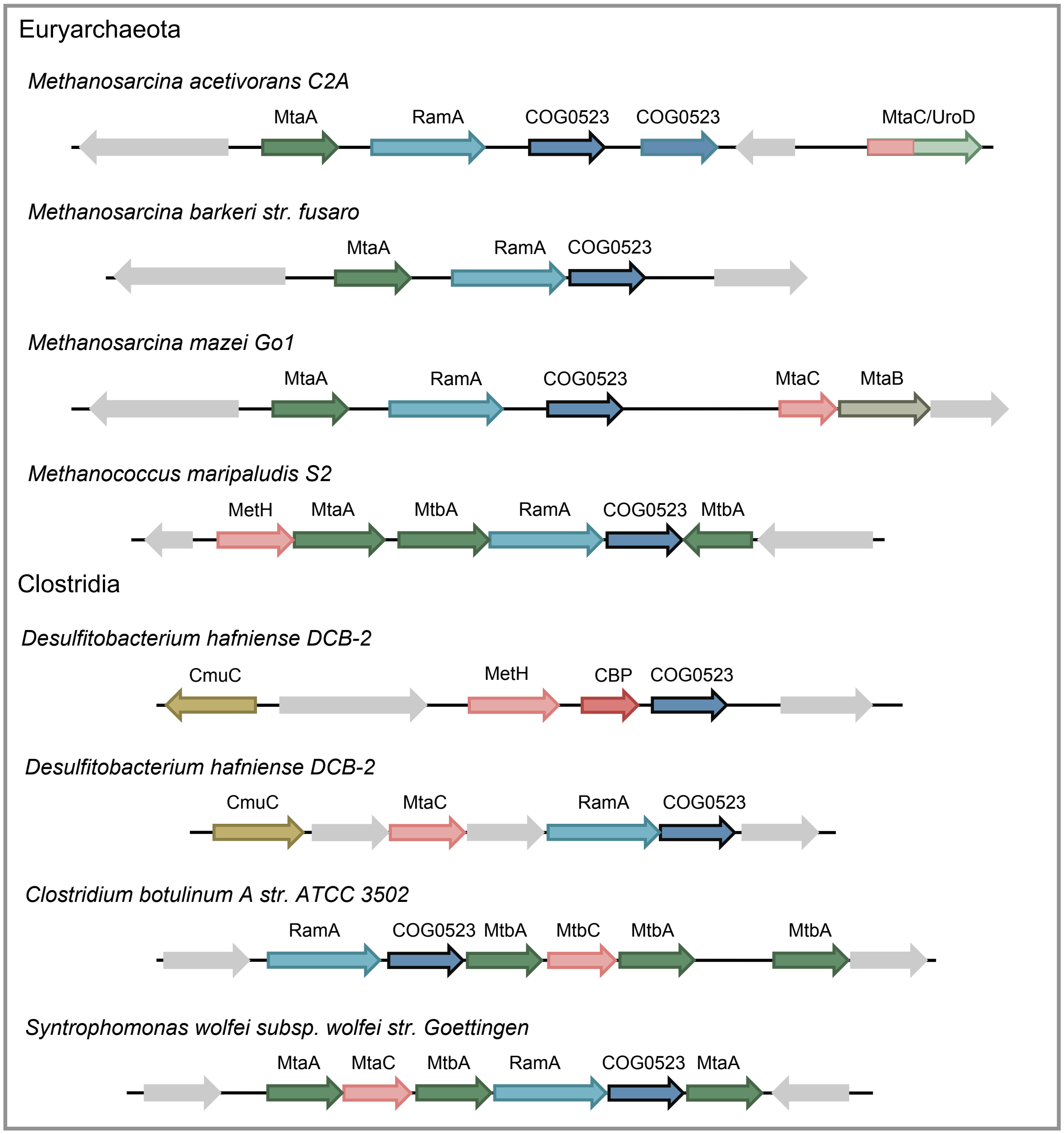


**Figure S12: Genome context of subgroup 13 members.**

Subgroup 13 co-localizes with genes encoding methyltransferases, corrinoid-binding proteins, and a protein responsible for corrinoid recycling. Abbreviations: CmuC, corrinoid methyltransferase-like; MetH, methyltetrahydrofolate:corrinoid/iron-sulfur protein methyltransferase; CBP, B12 binding domain of corrinoid proteins; MtaC/MtbC, corrinoid-binding protein; RamA, iron-sulfur protein that mediates the ATP-dependent reductive activation of Co(II) corrinoid to the Co(I) state; MtbA, Methylcobalamin:coenzyme M methyltransferase, methylaminie-specific; MtaA,

Methylcobalamin:coenzyme M methyltransferase, methanol-specific; MtaB, Methanol:corrinoid methyltransferase.

| Genome | Locus_tag |
|---|---|
| *Methanococcus maripaludis* S2 | MMP0833 |
| *Methanosarcina acetivorans* C2A | MA4382 MA4381 |
| *Methanosarcina barkeri* str. fusaro | Mbar_A1056 |
| *Methanosarcina mazei* Go1 | MM1072 |
| *Desulfitobacterium hafniense* DCB-2 | Dhaf_0359<br>Dhaf_3562 |
| *Clostridium botulinum* | CLB_1516 |
| *Syntrophomonas wolfei* subsp. *wolfei* str. Goettingen | Swol_0421 |

**Table S12. Genomes containing Subgroup 13 gene clusters.**

---

## Subgroup 14

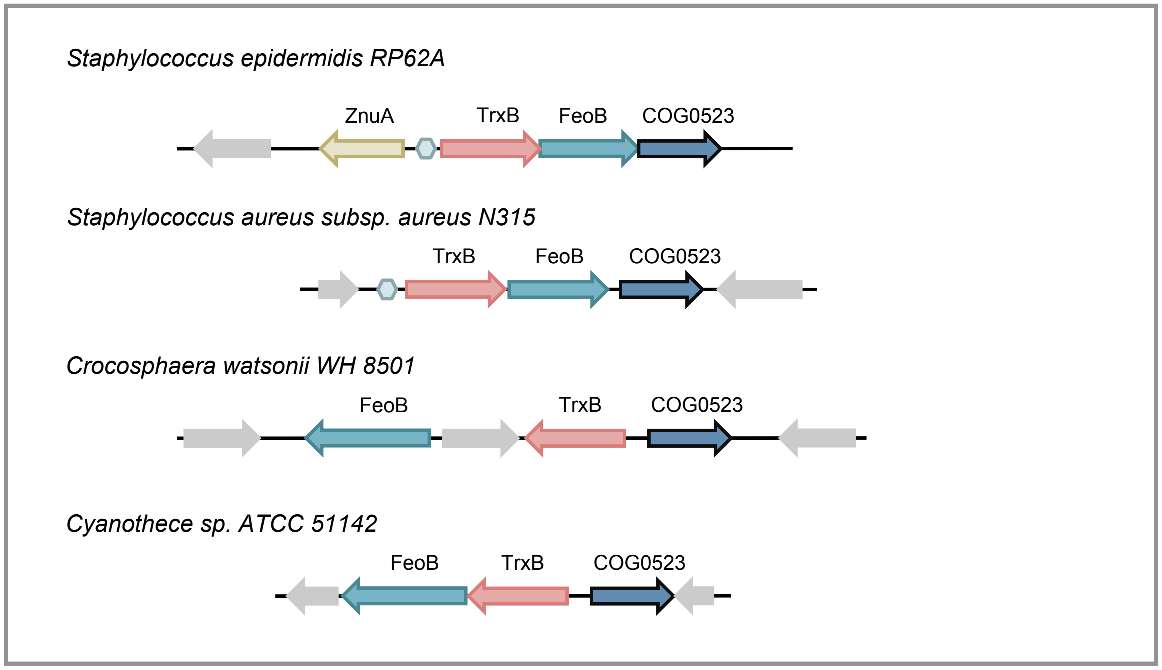


**Figure S13: Genome context of subgroup 14 members.**

Subgroup 14 co-localizes with genes encoding zinc ABC transport periplasmic-binding protein (ZnuA), thioredoxin reductase (TrxB), and iron transport protein (FeoB).

| Genome | Locus_tag |
|---|---|
| *Staphylococcus aureus* (13 genomes) | SAOUHSC_02901 (subsp. aureus NCTC 8325) |

| | |
|---|---|
| *Staphylococcus epidermidis* (2 genomes) | SE0188 (ATCC 12228) |
| *Crocosphaera watsonii* WH 8501 | CwatDRAFT_5580 |
| *Cyanothece sp. ATCC 51142* | Cce_4848 |

**Table S13. Genomes containing subgroup 14 gene clusters.**

**Subgroup 15**

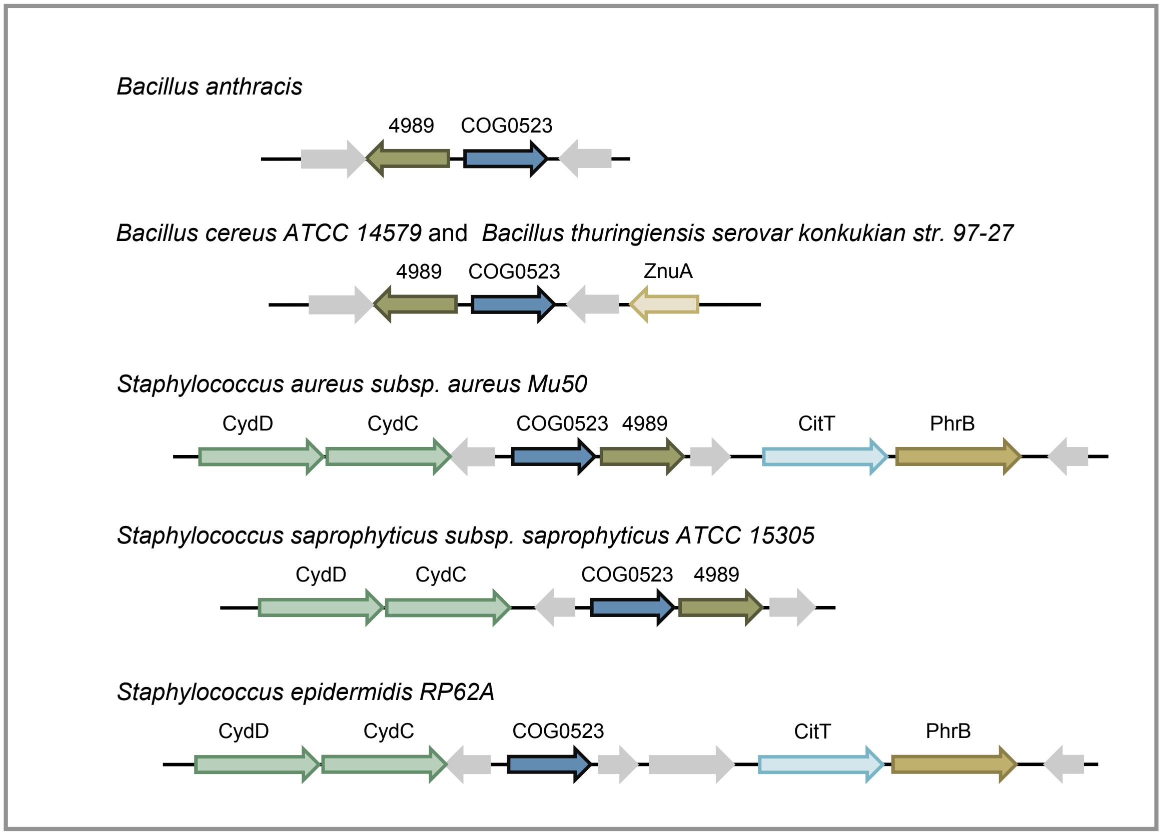


**Figure S14: Genome context of subgroup 15 members.**

Subgroup 15 co-localizes with genes encoding a putative oxidoreductase COG4989 (4989), ABC-type transport system involved in cytochrome bd biosynthesis (CydD and CydC), sodium/di- and tricarboxylate cotransporter (CitT), and deoxyribodipyrimidine photolyase (PhrB).

| Genome | Locus_tag |
|---|---|
| *Bacillus anthracis* (8 genomes) | BA2021 (str. Ames) |
| *Bacillus cereus* (5 genomes) | BCE2101 (str. ATCC 10987) |
| *Bacillus thuringiensis* (3 genomes) | BT9727_1849 (serovar konkukian str. 97-27) |
| *Bacillus weihenstephanensis* KBAB4 | BcerKBAB4_1883 |

| | |
|---|---|
| *Staphylococcus haemolyticus* JCSC1435 | SH2206 |
| *Staphylococcus aureus* subsp. *aureus* Mu50 | SAV0687 |
| *Staphylococcus saprophyticus* subsp. saprophyticus ATCC 15305 | SSP2031 |
| *Staphylococcus epidermidis* RP62A | SERP0344 |

**Table S14. Genomes containing subgroup 15 gene clusters.**

References


1. O'Mahony R, Doran J, Coffey L, Cahill OJ, Black GW, O'Reilly C: **Characterisation of the nitrile hydratase gene clusters of *Rhodococcus erythropolis* strains AJ270 and AJ300 and *Microbacterium* sp. AJ115 indicates horizontal gene transfer and reveals an insertion of IS1166.** *Anton Leeuw Int J G* 2005, **87**(3):221-232.
2. Nishiyama M, Horinouchi S, Kobayashi M, Nagasawa T, Yamada H, Beppu T: **Cloning and characterization of genes responsible for metabolism of nitrile compounds from *Pseudomonas chlororaphis* B23.** *J Bacteriol* 1991, **173**(8):2465-2472.
3. Kato Y, Yoshida S, Asano Y: **Polymerase chain reaction for identification of aldoxime dehydratase in aldoxime- or nitrile-degrading microorganisms.** *FEMS Microbiol Lett* 2005, **246**(2):243-249.
4. Kubáč D, Kaplan O, Elišáková V, Pátek M, Vejvoda V, Slámová K, Tóthová A, Lemaire M, Galienne E, Lutz-Wahl S *et al*: **Biotransformation of nitriles to amides using soluble and immobilized nitrile hydratase from *Rhodococcus erythropolis* A4**. *J Mol Catal B: Enzymatic* 2008, **50**(2-4):107-113.
5. Lu J, Zheng Y, Yamagishi H, Odaka M, Tsujimura M, Maeda M, Endo I: **Motif CXCC in nitrile hydratase activator is critical for NHase biogenesis in vivo.** *FEBS Lett* 2003, **553**(3):391-396.
6. Nojiri M, Yohda M, Odaka M, Matsushita Y, Tsujimura M, Yoshida T, Dohmae N, Takio K, Endo I: **Functional expression of nitrile hydratase in *Escherichia coli*: requirement of a nitrile hydratase activator and post-translational modification of a ligand cysteine.** *J Biochem* 1999, **125**(4):696-704.
7. Hashimoto Y, Nishiyama M, Horinouchi S, Beppu T: **Nitrile hydratase gene from *Rhodococcus* sp. N-774 requirement for its downstream region for efficient expression.** *Biosci Biotechnol Biochem* 1994, **58**(10):1859-1865.
8. Xie SX, Kato Y, Komeda H, Yoshida S, Asano Y: **A gene cluster responsible for alkylaldoxime metabolism coexisting with nitrile

**hydratase and amidase in *Rhodococcus globerulus* A-4.** *Biochemistry* 2003, **42**(41):12056-12066.

9. Thöny B, Auerbach G, Blau N: **Tetrahydrobiopterin biosynthesis, regeneration and functions.** *Biochem J* 2000, **347 Pt 1**:1-16.
10. Naponelli V, Noiriel A, Ziemak MJ, Beverley SM, Lye LF, Plume AM, Botella JR, Loizeau K, Ravanel S, Rébeillé F *et al*: **Phylogenomic and functional analysis of pterin-4a-carbinolamine dehydratase family (COG2154) proteins in plants and microorganisms.** *Plant Physiol* 2008, **146**(4):1515-1527.
11. Tanaka Y, Tsumoto K, Tanabe E, Yasutake Y, Sakai N, Yao M, Tanaka I, Kumagai I: **Crystal structure of the hypothetical protein ST2072 from *Sulfolobus tokodaii*.** *Proteins* 2005, **61**(4):1127-1131.
12. Morett E, Saab-Rincón G, Olvera L, Olvera M, Flores H, Grande R: **Sensitive genome-wide screen for low secondary enzymatic activities: the YjbQ family shows thiamin phosphate synthase activity.** *J Mol Biol* 2008, **376**(3):839-853.
13. Hille R: **Molybdenum-containing hydroxylases.** *Arch Biochem Biophys* 2005, **433**(1):107-116.
