# Supplementary material for: A subset of the diverse COG0523 family of putative metal chaperones is linked to zinc homeostasis in all kingdoms of life
Source: BMC Genomics. 2009 Oct 12;10:470. doi: 10.1186/1471-2164-10-470 (PMC2770081; doi:10.1186/1471-2164-10-470)

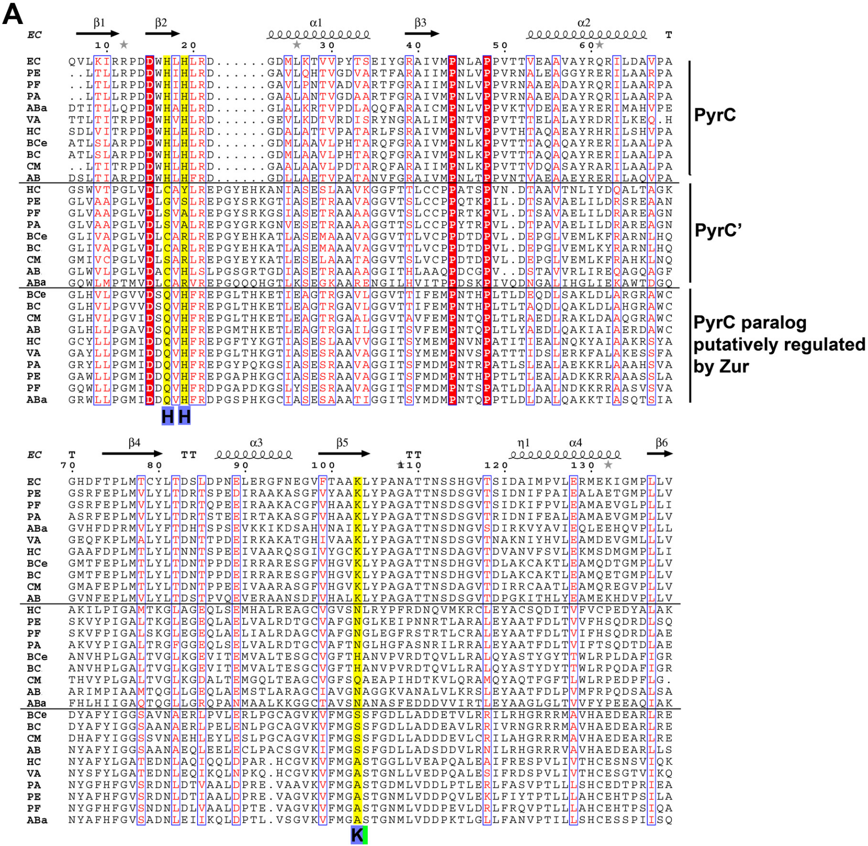

A
EC β1 β2 α1 β3 α2 T
10 20 30 40 50 60
EC QVLKIRRPDDWHLHLRD......GDMLKTVVPYTSEIYGRAIVMPNLAPPVTTVEAAVAYRQRILDAVPA
PE ..LTLLRPDDWHIHLRD......GAVLQHTVGDVARTFARAIIMPNLVPPVRNALEAGGYRERILAARPA
PF ..LTLLRPDDWHIHLRD......GAVLPNTVADVARTFGRAIIMPNLVPPVRNAAEADAYRQRILAARPA
PA ..LTLLRPDDWHIHLRD......GAALANTVGDAARTFGRAIVMPNLVPPVRNAAEADAYRQRILAARPA
ABa DTITLLQPDDWHAHLRD......GLALKRTVPDLAQQFARAICMPNLVPPVKTVDEAEAYRERIMAHVPE
VA TTLTITRPDDWHVHLRD......GEVLKDTVRDISRYNGRALIMPNTVPPVTTTELALAYRDRILKEQ.H
HC SDLVITRPDDWHLHLRD......GDALATTVPATARLFSRAIVMPNLVPPVTHTAQAEAYRHRILSHVPA
BCe ATLSLARPDDWHLHLRD......GDMLAAVLPHTARQFGRAIVMPNLKPPVTTTAQAQAYRERILAALPA
BC ATLSLARPDDWHLHLRD......GDMLAAVLPHTARQFGRAIVMPNLKPPVTTTAQAQAYRERILAALPA
CM ..LTITRPDDWHLHLRD......GAALAAVLPDTARQFARAIIMPNLKPPVTTVDQASAYRARILAALPA
AB DSLTIARPDDWHLHFRD......GDLLAETVPATANVFGRAIVMPNLTPPVNTVAEAAEYRERILAQVPA
HC GSWVTPGLVDLCAYLREPGYEHKANIASESLAAVKGGFTTLCCPPATSPVN.DTAAVTNLIYDQALTAGK
PE GLVAAPGLVDLGVSLREPGYSRKGTIASETRAAVAGGVTSLCCPPQTKPIL.DTSAVAELILDRSREAAN
PF GLVAAPGLVDLSVALREPGYSRKGSIASETRAAAAGGVTSLCCPPRTKPVL.DTSAVAELILDRAREAGN
PA GLVAAPGLVDLSVALREPGYGRKGNVESETRAAAAGGTTSLCCPPYTRPVL.DTPAVAELILDRAREAGN
BCe GLIVAPGLVDLCARLREPGYEHKATLASEMAAAVAGGVTTLVCPPDTDPVL.DEPGLVEMLKFRARNLHQ
BC GLIVAPGLVDLCARLREPGYEHKATLASEMAAAVAGGVTTLVCPPDTDPVL.DEPGLVEMLKYRARNLHQ
CM GMIVCPGLVDLSARLREPGYEYKATLESELAAATAGGVTSLVCPPDTDPVL.DEPGLVEMLKFRAHKLNQ
AB GLWVLPGLVDACVHLSLPGSGRTGDIASETRAAAAGGITHLAAQPDCGPV..DSTAVVRLIREQAGQAGF
ABa GQWLMPTMVDLCARVREPGQQQHGTLKSEGKAAREN GILHVITPPDSKPIVQDNGALIHGLIEKAWTDGQ
BCe GLHVLPGVVDSQVHFREPGLTHKETIEAGTRGAVLGGVTTIFEMPNTHPLTLDEQDLSAKLDLARGRAWC
BC GLHVLPGVVDSQVHFREPGLTHKETLEAGTRGAVLGGVTTIFEMPNTHPLTLTAQDLQAKLDLAHGRAWC
CM GLHVLPGVIDSQVHFREPGLTHKETLEAGTRGAALGGVTAVFEMPNTQPLTLRAEDLRAKLDAAQGRAWC
AB GLHLLPGAVDSQVHFREPGMTHKETLEAGTRGAILGGITSVFEMPNTQPLTLYAEDLQAKIAIAERDAWC
HC GCYLLPGMIDDQVHFREPGFTYKGTIASESLAAVVGGITSYMEMPNVNPATITIEALNQKYAIAAKRSYA
VA GAYLLPGMIDDQVHFREPGLTHKGTIASESRAAVAGGITSYMEMPNVSPATTTIDSLERKFALAKESSFA
PA GRYLLPGMIDDQVHFREPGYPQKGSIASESRAAVAGGITSFMDMPNTRPATLSLEALAEKKRLAAAHSVA
PE GAWLLPGMIDDQVHFREPGAPHKGCIASESRAAVAGGITSFMDMPNTHPPTLTLEALADKKRRAAASSLA
PF GQWLLPGMIDDQVHFRDPGAPDKGSLYSESRAAVAGGITSFMDMPNTSPATLDLAALNDKKRRAASVSVA
ABa GRWLLPGMIDDQVHFRDPGSPHKGCIASESAAATIGGITSYMDMPNTQPPTLDLDALQAKKTIASQTSIA
H H
PyrC
PyrC’
PyrC paralog putatively regulated by Zur
EC T β4 TT α3 β5 TT η1 α4 β6
70 80 90 100 110 120 130
EC GHDFTPLMTCYLTDSLDPNELERGFNEGVFTAAKLYPANATTNSSHGVTSIDAIMPVLERMEKIGMPLLV
PE GSRFEPLMVLYLTDRTSPEDIRAAKASGFVYAAKLYPAGATTNSDSGVTSIDNIFPAIEALAETGMPLLV
PF GSRFEPLMVLYLTDRTQPEEIRAAKACGFVHAAKLYPAGATTNSDSGVTSIDKIFPVLEAMAEVGLPLLI
PA ASRFEPLMVLYLTDRTSTEEIRTAKASGFVHAAKLYPAGATTNSDSGVTRIDNIFEALEAMAEVGMPLLV
ABa GVHFDPRMVLYFTDHTSPSEVKKIKDSAHVNAIKLYPAGATTNSDNGVSDIRKVYAVIEQLEEHQVPLLL
VA GEQFKPLMALYLTDNTTPDEIRKAKATGHIVAAKLYPAGATTNSDSGVTNAKNIYHVLEAMDEVGMLLLV
HC GAAFDPLMTLYLTNNTSPEEIVAARQSGIVYGCKLYPAGATTNSDAGVTDVANVFSVLEKMSDMGMPLLI
BCe GMTFEPLMTLYLTDNTPPDEIRRARESGFVHGVKLYPAGATTNSDHGVTDLAKCAKTLEAMQDTGMPLLV
BC GMTFEPLMTLYLTDNTPPDEIRRARESGFVHGVKLYPAGATTNSDHGVTDLAKCAKTLEAMQETGMPLLV
CM GMAFEPLMTLYLTDNTPPEEIVAARASGFVHGVKLYPAGATTNSDAGVTDIRRCAATLEAMQREGVPLLV
AB GVNFEPLMVLYLTDSTPVQEVERAANSDFVHALKLYPAGATTNSDSGVTDPGKITHLYEAMEKHDVPLLV
HC AKILPIGAMTKGLAGEQLSEMHALREAGCVGVSNLRYPFRDNQVMKRCLEYACSQDITVFVCPEDYALAK
PE SKVYPIGALTKGLEGEQLAELVALRDTGCVAFGNGLKEIPNNRTLARALEYAATFDLTVVFHSQDRDLSQ
PF SKVFPIGALSKGLEGEQLAELIALRDAGCVAFGNGLEGFRSTRTLCRALEYAATFDLTVIFHSQDRDLAE
PA AKVYPIGALTRGFGGEQLSELVALRDTGCVAFTNGLHGFASNRILRRALEYAATFDLTVIFTSQDTDLAE
BCe ANVHPLGALTVGLKGEVITEMVALTESGCVGFTHANVPVRDTQVLLRALQYASTYGYTTWLRPLDAFIGR
BC ANVHPLGALTVGLKGEVITEMVALTESGCVGFTHANVPVRDTQVLLRALQYASTYDYTTWLRPQDAFIGR
CM THVYPLGALTVGLKGDALTEMGQLTEAGCVGFSQAEAPIHDTKVLQRAMQYAQTFGFTLWLRPEDPFLG.
AB ARIMPIAAMTQGLLGEQLAEMSTLARAGCIAVGNAGGKVANALVLKRSLEYAATFDLPVMFRPQDSALSA
ABa FHLHIIGAQTQGLLGRQPANMAALKKGGCTAVSNANASFEDDDVVIRTLEYAAGLGLTVVFYPEEAQIAK
BCe DYAFYIGGSAVNAERLPVLERLPGCAGVKVFMGSSFGDLLADDETVLRRILRHGRRRMAVHAEDEARLRE
BC DYAFYIGGSAANAERLPELENLPGCAGVKVFMGSSFGDLLADDEAVLRRIVRNGRRRMAVHAEDEARLRE
CM DHAFYIGGSSVNAEHLEYLESLPGCAGVKIFMGSSFGDLLADDDEVLCRILAHGRRRMAVHAEDEARLRE
AB NYAFYIGGSAANAEQLEELECLPACSGVKIFMGSSFGDLLADSDDVLRNILRHGRRRVAVHAEDEARLLS
HC NYAFYLGATEDNLAQIQQLDPAR.HCGVKVFMGASTGGLLVEAPQALEAIFRESPVLIVTHCESNSVIQK
VA NYSFYLGATEDNLEQIKQLNPKQ.HCGVKVFMGASTGNLLVEDPQALESIFRDSPVLIVTHCESGTVIKQ
PA NYGFHFGVSRDNLDTVAALDPRE.VAAVKVFMGASTGDMLVDDLPTLERLFASVPTLLLSHCEDTPRIEA
PE NYAFHFGVSRDNLDTIAALDPRE.VAGVKVFMGASTGNMLVDDPQVLEKLFIYTPTLLLAHCEHTPSILA
PF NYGFHFGVSNDNLDLVAALDPTE.VAGVKVFMGASTGNMLVDDPEVLDRLFRQVPTLLLAHCEHTPSIQA
ABa NYAFHFGVSADNLEIIKQLDPTL.VSGVKVFMGASTGNMLVDDPKTLGLLFANVPTILLSHCESSPIISQ
K

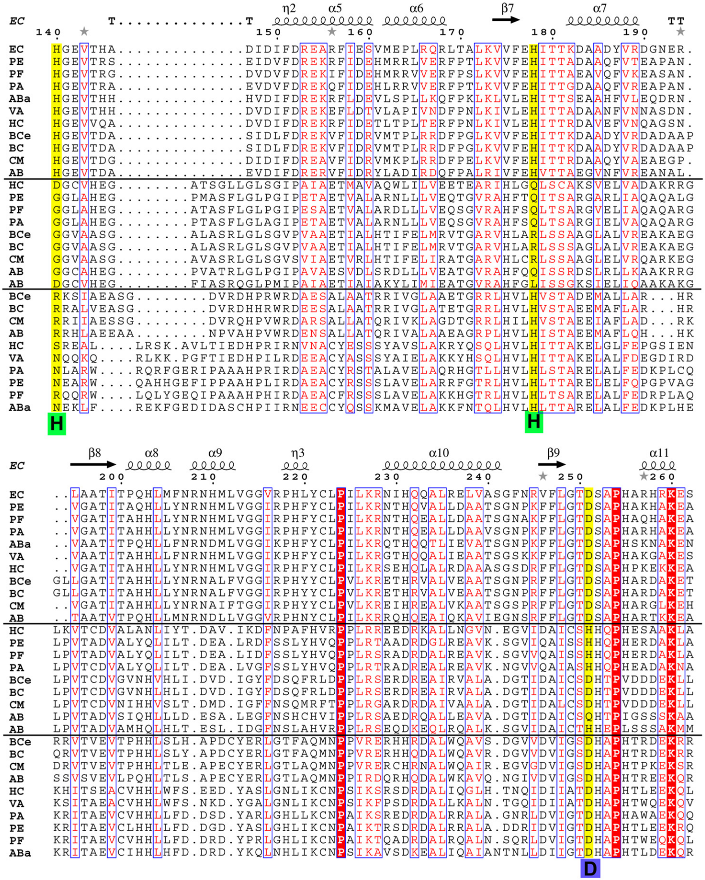

EC
η2 α5 α6 β7 α7
T..............T
TT
140 150 160 170 180 190
EC HGEVTHA..............DIDIFDREARFIESVMEPLRQRLTALKVVFEHITTKDAADYVRDGNER.
PE HGEVTRS..............EIDVFDREKRFIDEHMRRLVERFPTLKVVFEHITTADAAQFVTEAPAN.
PF HGEVTRG..............DVDVFDREKIFIDEHMRRVVERFPSLKVVFEHITTAEAVQFVKEASAN.
PA HGEVTRA..............EVDVFDREKQFIDEHLRRVVERFPTLKVVFEHITTGDAAQFVREAPAN.
ABa HGEVTHH..............HVDIFDREKRFLDEVLSPLLKQFPKLKLVLEHITTSEAAHFVLEQDRN.
VA HGEVTHH..............DVDIFDREKEFLDTVLAPIVNDFPNLKIVLEHITTADAANFVNNASDN.
HC HGEVVQA..............DVDIFDREKRFIDETLTPLTERFPNLKIVLEHITTRDAVEFVNQAGSN.
BCe HGEVTDA..............SIDLFDREKVFIDRVMTPLRRDFPGLKVVFEHITTKDAADYVRDADAAP
BC HGEVTDA..............SIDLFDREKVFIDRVMTPLRRDFPGLKVVFEHITTKDAADYVRDADAAP
CM HGEVTDG..............DIDIFDREAVFIDRVMKPLRRDFPELKVVFEHITTRDAAQYVAEAEGP.
AB HGEVTDA..............EVDIFDRERVFIDRYLADIRDQFPALRIVFEHVTTAEGVNFVREANAL.
HC DGCVHEG........ATSGLLGLSGIPAIAETMAVAQWLILVEETEARIHLGQLSCAKSVELVADAKRRG
PE GGLAHEG........PMASFLGLPGIPETAETVALARNLLVEQTGVRAHFTQITSARGARLIAQAQALG
PF GGLAHEG........ATASFLGLPGIPESAETVALARDLLVEQSGVRAHFSQLTSARGVELIAQAQARG
PA GGLAHEG........PTASFLGLAGIPETAETVALARNLLVEQSGVRAHFSQLTSARGIELVAQAQARG
BCe GGVAASG........ALASRLGLSGVPVAAETIALHTIFELMRVTGARVHLARLSSAAGLALVREAKAEG
BC GGVAASG........ALASRLGLSGVPVAAETIALHTIFELMRVTGARVHLARLSSSAGLALVREAKAEG
CM GGVAASG........AVASRLGLSGVSVIAETVRLHTIFELIRATGARVHLCRLSSAAGLELVRQARKEG
AB GGCAHEG........PVATRLGLPGIPAVAESVDLSRDLLVEATGVRAHFQQISSRDSLRLLKAAKRRG
AB DGCVHEG........FIASRQGLPMIPAIAETIAIAKYLIMIEATGVRAHFGLISSGKSIELIQAAKAKG
BCe RKSIAEASG........DVRDHPRWRDAESALAATRRIVGLAAETGRRLHVLHVSTADEMALLAR...HR
BC RRALVEASG........DVRDHHRWRDEESALAATRRIVGLATETGRRLHVLHVSTAEEMAFLAR...HR
CM RRIIAESSG........DVRQHPVWRDARSALLATQRIVKLAGDTGRRLHILHVSTAEEIAFLAD...RK
AB RRHLAEEAA........NPVAHPVWRDENSALLATQRIVALAAEAGRPLHVLHISTAEEMAFLQH...HK
HC SREAL....LRSK.AVLTIEDHPRIRNVNACYESSSYAVSLAKRYQSQLHVLHITTAKELGLFEPGSIEN
VA NQQKQ....RLKK.PGFTIEDHPILRDEEACYASSSYAIELAKKYHSQLHVLHITTAKELALFDEGDIRD
PA NLARW....RQRFGERIPAAAHPIRRDAEACYRSTALAVELAQRHGTLLHVLHLSTARELALFEDKPLCQ
PE NEARW....QAHHGEFIPPAAHPLIRDAEACYRSSSLAVELARRFGTRLHVLHLTTARELELFQPGPVAG
PF RQQRW....LQLYGEQIPAAAHPHIRDAEACFRSSSLAVELAKKHGTRLHVLHLTSARELALFEDRPLAQ
ABa NEKLF....REKFGEDIDASCHPIIRNEECCYQSSKMAVELAKKFNTQLHVLHLTTARELALFEDKPLHE
H
H
EC
β8 α8 α9 η3 α10 β9 α11
200 210 220 230 240 250 260
EC ..LAATITPQHLMFNRNHMLVGGVRPHLYCLPILKRNIHQQALRELVASGFNRVFLGTDSAPHARHRKES
PE ..VGATITAQHLLYNRNHMLVGGIRPHFYCLPILKRNTHQVALLDAATSGNPKFFLGTDSAPHAKHAKEA
PF ..VGATITAHHLLYNRNHMLVGGIRPHFYCLPILKRNTHQEALLDAATSGNAKFFLGTDSAPHAQHAKEA
PA ..VGATITAHHLLYNRNHMLVGGIRPHFYCLPILKRNTHQEALLDAAVSGNPKFFLGTDSAPHARHAKEA
ABa ..VAATITPQHLLFNRNDMLVGGIKPHFYCLPILKRQTHQQTLIEVATSGNPKFFLGTDSAPHSKNAKEN
VA ..VAATITAHHLLFNRNHMLVGGIKPHFYCLPILKRGTHQQALIEAATSGSKKFFLGTDSAPHAKGAKES
HC ..VGATITAHHLLYNRNHMLVGGIRPHFYCLPILKRSEHQLALRDAAASGSDRFFLGTDSAPHPKEKKEA
BCe GLLGATITAHHLLYNRNALFVGGIRPHYYCLPVLKRETHRVALVEAATSGNPRFFLGTDSAPHARDAKET
BC GLLGATITAHHLLYNRNALFVGGIRPHYYCLPVLKRETHRVALVEAATSGNPRFFLGTDSAPHARDAKET
CM ..VGATITAHHLLYNRNAIFTGGIRPHYYCLPVLKREIHREALVKAATSGSPRFFLGTDSAPHARGLKEH
AB ..TAATVTPQHLLMNRNDLLVGGVRPHNYCLPILKRRQHQEAIQKAVIEGNSRFFLGTDSAPHARTKKEA
HC LKVTCDVALANLIYT.DAV.IKDFNPAFHVRPPLRREEDRKALLNGVN.EGVIDAICSHHQPHESAAKLA
PE LPVTADVALYQLILT.DEA.LRDFSSLYHVQPPLRTAADRDGLREAVK.SGVIQAISSHHQPHERDAKLA
PF LPVTADVALYQLILT.DEA.LIDFSSLYHVQPPLRSRADRDALREAVK.SGVVQAISSHHQPHERDAKLA
PA LPVTCDVALYQLILT.DEA.LVGFSSLYHVQPPLRTRADREALREAVK.NGVVQAIASHHQPHEADAKNA
BCe LPVTCDVGVNHVHLI.DVD.IGYFDSQFRLDPPLRSERDREAIRVALA.DGTIDAICSDHTPVDDDEKLL
BC LPVTCDVGVNHLHLI.DVD.IGYFDSQFRLDPPLRSERDREAIRVALA.DGTIDAICSDHTPVDDDEKLL
CM LPVTCDVNIHHVSLT.DMD.IGFFNSQMRFTPPLRGARDRDAIVALA.DGTIDALCSDHTPVDDDEKLL
AB LPVTADVSIQHLLLD.ESA.LEGFNSHCHVIPPLRSAEDRQALLEAVA.DGTIDAICSQHTPIGSSSKAA
AB LPVTADVAMHQLHLT.ESL.IDGFNSLAHVRPPLRSEQDKEQLRQALK.SGVIDAICTHHEPLSSSAKMM
BCe RRVTVEVTPHHLSLH.APDCYERLGTFAQMNPPVRERHHRDALWQAVS.DGVVDVIGSDHAPHTRDEKRR
BC QRVTVEVTPHHLSLY.APDCYERLGTFAQMNPPVRERHHQDALWQAVR.DGVVDVIGSDHAPHTRDEKRR
CM DRVTVEVTPHHLTLE.APECYERLGTLAQMNPPVRERCHRNALWQAIR.EGVGDVIGSDHAPHTPKEKSR
AB SSVSVEVLPQHLTLS.APECYERLGTLAQMNPPIRDQRHQDALWKAVQ.NGIVDVIGSDHAPHTREEKQR
HC KHITSEACVHHLWFS.EED.YASLGNLIKCNPSIKSRSDRDALIQGLH.TNQIDIIATDHAPHTLEEKQL
VA KSITAEACVHHLWFS.NKD.YGALGNLIKCNPSIKFPSDRDALLKALN.TGQIDIIATDHAPHTWQEKQV
PA KRITAEVCVHHLLFD.DSD.YARLGHLLKCNPAIKSREDRDALRRALA.GNRLDVIGTDHAPHAWAEKQQ
PE KRITAEVCLHHLLFD.DGD.YASLGHLIKCNPAIKTRSDRDALRRALA.DDRIDVIGTDHAPHTLEEKRQ
PF KRITAEVCVHHLLFD.DRD.YPRLGHLIKCNPAIKTQADRDALRQALN.SQRLDVIGTDHAPHTWEQKQL
ABa KRITAEVCIHHLHFD.DRD.YKQLNHLIKCNPSIKAVSDKEALIQAIANTNLLDIIGTDHAPHTLDEKQR
D

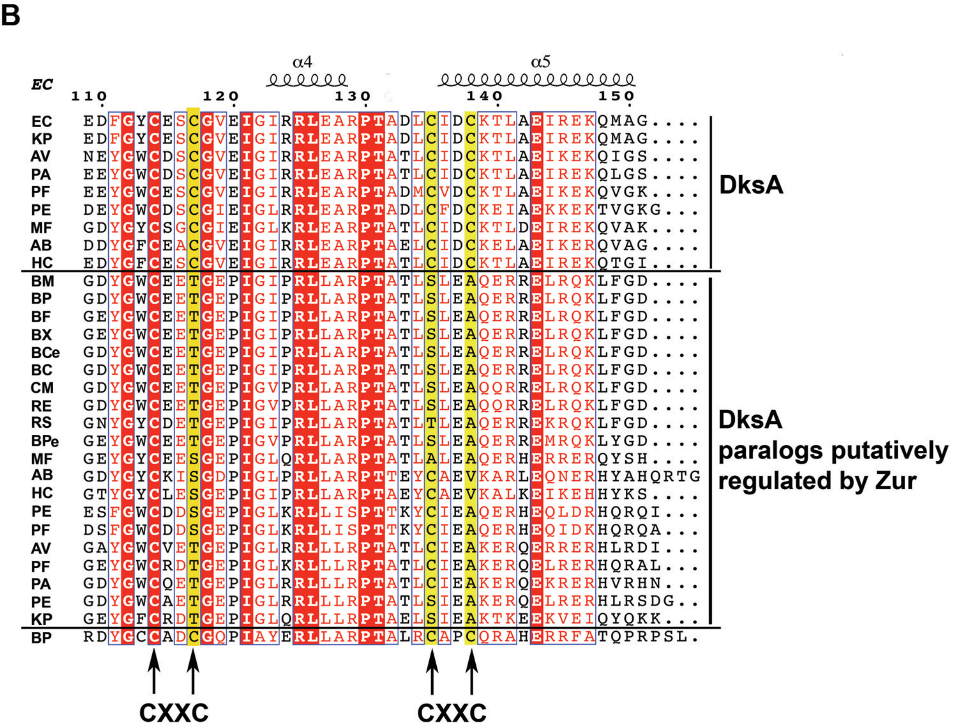

B
α4
α5
EC
110
120
130
140
150
DksA
DksA paralogs putatively regulated by Zur
CXXC
CXXC

C

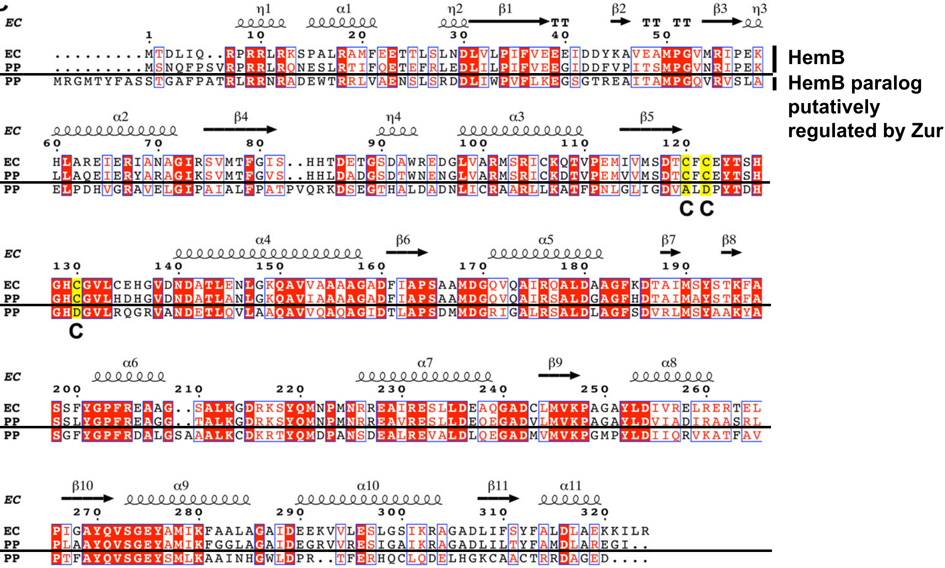

Supplement: Additional file 6 — Sequence analysis of PyrC, DksA and HemB paralogs. Alignments of protein sequences encoded by genomes from Figure 4. A, Alignment of the three PyrC paralogs. The residues that chelate the α-metal are highlighted in blue while the β-metal ligands are highlighted in green. Lys102 serves as a ligand for both metals ions. B, DksA paralogs. C, HemB paralogs. Secondary structure as determined from the crystal structure of the Escherichia coli homolog in each case is given (PDB identifiers: 1J79 (PyrC), 1TJL (DksA), 1L6S (HemB)). For PyrC and DksA, the alignments show only the portion of the alignment containing the zinc binding residues. Columns containing the zinc chelating residues as determined from the crystal structure are highlighted in yellow. Genome abbreviations: EC, Escherichia coli; PE, Pseudomonas entomophila L48; PF, Pseudomonas fluorescens Pf-5; PA, Pseudomonas aeruginosa PAO1; ABa, Acinetobacter baylyi ADP1; VA, Vibrio alginolyticus 12G01; HC, Hahella chejuensis KCTC 2396; BCe, Burkholderia cenocepacia AU 1054; BC, Burkholderia cepacia R18194; CM, Cupriavidus metallidurans CH34l; AB, Alcanivorax borkumensis SK2; KP, Klebsiella pneumoniae MGH 78578 (on virulence plasmid pLVPK); AV, Azotobacter vinelandii; BPe, Bordetella pertussis Tohama I; BF, Burkholderia fungorum; BM, Burkholderia mallei ATCC 23344; BX, Burkholderia xenovorans LB400; BP, Burkholderia pseudomallei 1710b; RE, Ralstonia eutropha JMP134; RS, Ralstonia solanacearum GMI1000; MF, Methylobacillus flagelatus KT. [file 1471-2164-10-470-S6.PDF]
