# Supplementary material for: A subset of the diverse COG0523 family of putative metal chaperones is linked to zinc homeostasis in all kingdoms of life
Source: BMC Genomics. 2009 Oct 12;10:470. doi: 10.1186/1471-2164-10-470 (PMC2770081; doi:10.1186/1471-2164-10-470)
Supplement: Additional file 9 — Sequence logos for DNA-binding motifs for candidate Zinc regulators. The taxonomy-specific DNA motif logos were constructed using Zur- and ZntR-binding sites indentified for COG0523 and other zinc-responsive genes described in the Additional Files 5 and 7. [file 1471-2164-10-470-S9.PDF]

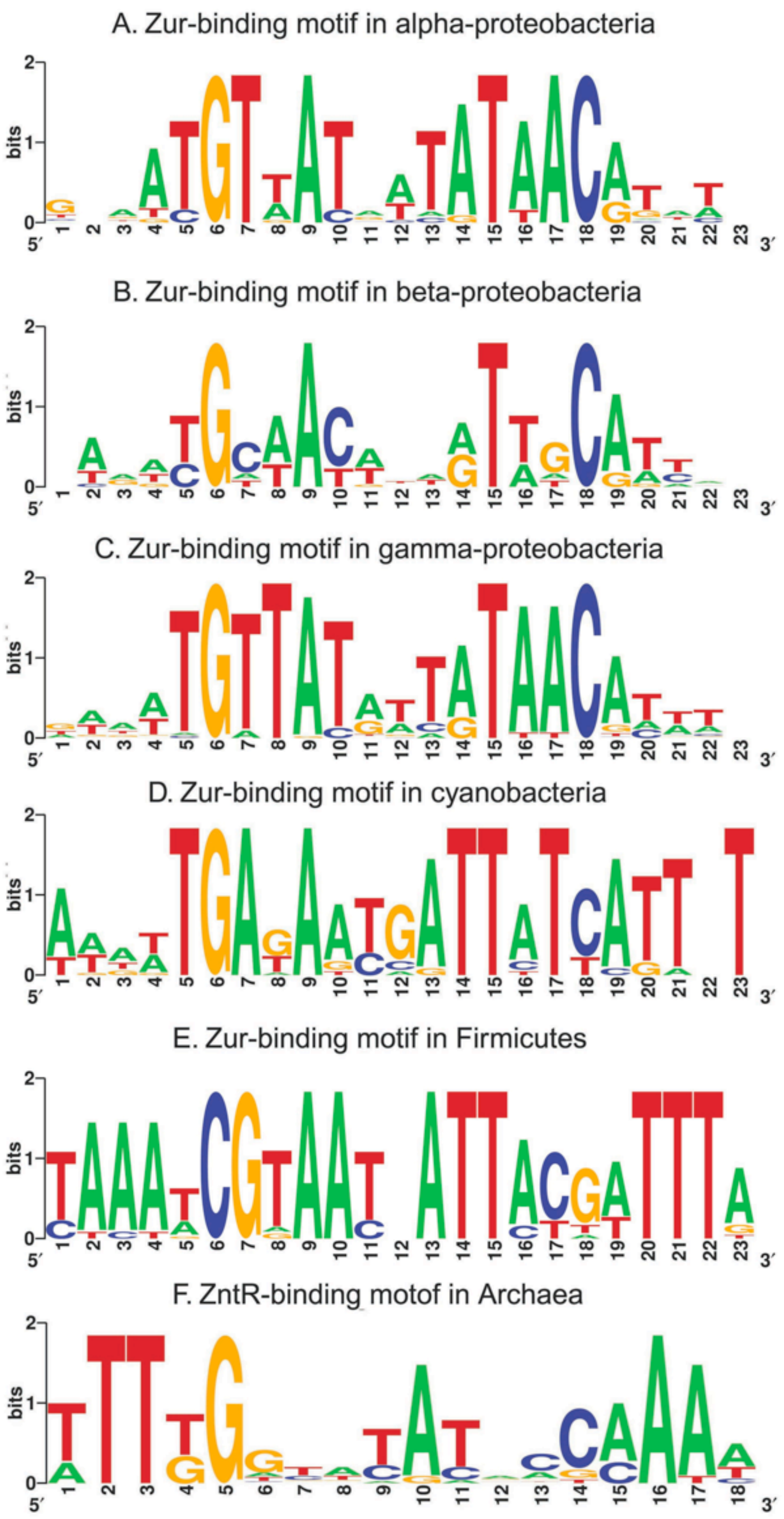

A. Zur-binding motif in alpha-proteobacteria
B. Zur-binding motif in beta-proteobacteria
C. Zur-binding motif in gamma-proteobacteria
D. Zur-binding motif in cyanobacteria
E. Zur-binding motif in Firmicutes
F. ZntR-binding motof in Archaea
bits
5′
3′
