# Supplementary material for: A subset of the diverse COG0523 family of putative metal chaperones is linked to zinc homeostasis in all kingdoms of life
Source: BMC Genomics. 2009 Oct 12;10:470. doi: 10.1186/1471-2164-10-470 (PMC2770081; doi:10.1186/1471-2164-10-470)
Supplement: Additional file 10 — Primers used in transcription analyses. Sequences for primers used in the transcription analysis of A. baylyi and C. reinhardtii COG0523 homologs. [file 1471-2164-10-470-S10.PDF]

Protein ID	Forward Primer	Reverse Primer	
Acinetobacter baylyi ADP1			
ACIAD1025	CGTATTGGCCTGTCCTGTTT	ATGAAGAGGCATGGGTTCAG	
ACIAD1614	CGCACTGTATACCCATGACG	TTAACTCCACACGCATTCCA	
ACIAD1741	TACCGTGTCTTGCCATACCA	AAACGCTTGGCAGAAGAAAA	
Chlamydomonas reinhardtii 2137			efficiency
123019	ACGAGTGGCCTGAGGACGAG	ATTGAGCCCCTGTCCGATGA	104 %
117458	ACGTCTGGGGTGCATTCCTC	GGGATGGGGCAGGTATGTGT	98 %
105568	CAAGGGCTTCATGTGGCTGA	CCTCGTCCCGGATCTCAAAG	99 %
122261	GCGATCAAGGGCTCAGACGA	TGCCGATGAACACCACCTTG	101 %
106748	GCTCTGATCGTGGGCTCTCC	ATGGTCTCGTGCTGGGGTTC	101 %
195946	GAGGCGCGACGTTAGGAAAG	GAACAAGCAGCCGCACCAAG	96 %
106402	ATGCTGGACGGATGCCTGTT	CTCCCACTCCCCCTCATCCT	98 %
101629	CCTCGGCAGGTTCAAGTCGT	GGTCGGAGTTCGCCACAAAG	101 %
143868	CAGGGTGCAGAGCTTGTTGG	CCCCCACGCCTTTCGCAGGA	95 %
73360	ATTGCGTGGTGGAGAGCACA	CGAGGGGCAGTAGCAGTAGCA	103 %
