# Supplementary material for: A subset of the diverse COG0523 family of putative metal chaperones is linked to zinc homeostasis in all kingdoms of life
Source: BMC Genomics. 2009 Oct 12;10:470. doi: 10.1186/1471-2164-10-470 (PMC2770081; doi:10.1186/1471-2164-10-470)
Supplement: Additional file 11 — MIQE checklist for C. reinhardtii qRT-PCR. [file 1471-2164-10-470-S11.DOC]

| **EXPERIMENTAL DESIGN** |
| --- |
| Definition of experimental and control groups |
| Number within each group |
| Assay carried out by core lab or investigator's lab? |
| Acknowledgement of authors' contributions |
| **SAMPLE** |
| Description |
| Volume/mass of sample processed |
| Microdissection or macrodissection |
| Processing procedure |
| If frozen - how and how quickly? |
| If fixed - with what, how quickly? |
| Sample storage conditions and duration (especially for FFPE samples) |

Experimental design is provided in the material and method section. RNA was isolated from three different cultures at each condition. The control (metal replete) was generated by adding a known metal concentration back to one culture prior to cell growth. Assays were carried out in the investigator’s lab.

In general, about 100 ml of culture was collected and RNA was isolated immediately.

| **NUCLEIC ACID EXTRACTION** |
| --- |
| Procedure and/or instrumentation |
| Name of kit and details of any modifications |
| Source of additional reagents used |
| Details of DNase or RNAse treatment |
| Contamination assessment (DNA or RNA) |
| Nucleic acid quantification |
| Instrument and method |
| Purity (A260/A280) |
| Yield |
| RNA integrity method/instrument |
| RIN/RQI or Cq of 3' and 5' transcripts |
| Electrophoresis traces |
| Inhibition testing (Cq dilutions, spike or other) |

The RNA isolation procedure is described in Hill et al 1991, no kit was used to isolate RNA. To remove any remaining DNA traces, 40µg RNA was treated with 1unit of RQ1 DNAse (Promega, Cat#M6101) in a 100 µl volume. All following procedures were performed according to the manufacturer’s instructions except that an RNA precipitation was performed after heat inactivation of the enzyme. Contamination was assessed by a no RT control or direct use of treated RNA in the qPCR reaction; additionally, the control gene used for qPCR (e.g. *CBLP*) is flanking an intron and since a melting curve is performed as standard, a contamination would be visible as additional peak. The precipitated RNA was resuspended in water. A 1:100 dilution was measured with the Lambda 25 from Perkin-Elmer. The A260/280 ratio is generally between 1.9 and 2.0, exact values for each RNA can be provided upon request. The yield is about 1 mg of RNA per 100 ml culture.

RNA integrity (RIN/RQI or Cq of 3' and 5' transcripts, Electrophoresis traces) and inhibition testing was not performed. However, the position of the threshold is standardized and the Cq values for the control gene *CBLP* was within 1 cycle for all cDNAs used.

(Hill, K., Li, H.H., Singer, J., Merchant, S. (1991) Isolation and Structural Characterization of the *Chlamydomonas reinhardtii* Gene for Cyt c6: Analysis of the Kinetics and Metal Specificity of its Cu-responsive Expression. J. Biol. Chem. 266:15060-15067.)

| **REVERSE TRANSCRIPTION** |
| --- |
| Complete reaction conditions |
| Amount of RNA and reaction volume |
| Priming oligonucleotide (if using GSP) and concentration |
| Reverse transcriptase and concentration |
| Temperature and time |
| Manufacturer of reagents and catalogue numbers |
| Cqs with and without RT |
| Storage conditions of cDNA |

M-MLV Reverse Transcriptase from Invitrogen (Cat#28025-021; 200 U/ µl) was used for the generation of first strand cDNA in a 40 µl reaction volume. 10 µl of RNA (0.5 mg/ml), 2 µl of oligo(dT)_18_ (500 µg/ml) and 2 µl dNTP mix (NEB, Cat# N0447L) were incubated at 70°C for 10 min and quick chilled on ice. All other steps were performed per manufacturer’s instructions except that the incubation time at 37°C was increased from 50 to 90 min. For most cDNAs, the *CBLP* amplicon was used for the estimation of contamination and in this case the Cqs with RT are about 13, without RT no amplification was detected. cDNA was stored in low adhesion tubes at -20°C.

| **qPCR TARGET INFORMATION** |
| --- |
| If multiplex, efficiency and LOD of each assay. |
| Sequence accession number |
| Location of amplicon |
| Amplicon length |
| *In silico* specificity screen (BLAST, etc) |
| Pseudogenes, retropseudogenes or other homologs? |
| Sequence alignment |
| Secondary structure analysis of amplicon |
| Location of each primer by exon or intron (if applicable) |
| What splice variants are targeted? |

Multiplex qPCR was not performed. Sequence accession numbers are

XP_001702424 (123019), XP_001692771 (117458), XP_001697680 (105568), XP_001700675 (122261), XP_001699301 (106748), XP_001692085 (195946), XP_001699037 (106402), XP_001689783 (101629), XP_001703275 (143868), XP_001702424 (73360). Amplicon length is included in table under qPCR validation. *In silico* screen were performed with NCBI Blast and can be obtained from above web side. Primer were designed in exons or UTR regions close to the 3’end of the gene. No splice variants were targeted.

| **qPCR OLIGONUCLEOTIDES** |
| --- |
| Primer sequences |
| RTPrimerDB Identification Number |
| Probe sequences |
| Location and identity of any modifications |
| Manufacturer of oligonucleotides |
| Purification method |

Primer sequence are included in the manuscript as supplemental file 10. No modifications were used. Primers were purchase from Operon Biotechnologies (now Eurofins MWG Operon) and are salt-free.

| **qPCR PROTOCOL** |
| --- |
| Complete reaction conditions |
| Reaction volume and amount of cDNA/DNA |
| Primer, (probe), Mg++ and dNTP concentrations |
| Polymerase identity and concentration |
| Buffer/kit identity and manufacturer |
| Exact chemical constitution of the buffer |
| Additives (SYBR Green I, DMSO, etc.) |
| Manufacturer of plates/tubes and catalog number |
| Complete thermocycling parameters |
| Reaction setup (manual/robotic) |
| Manufacturer of qPCR instrument |

Each qPCR reaction had a 20 µl reaction volume containing:

cDNA corresponding to 100ng input RNA

150 nM of each forward and reverse primer

2 mM Mg^2+^

4 mM dNTP

about 2 u Taq*

1 x exTAQ buffer (TaKaRA, Cat#9152A)

1 x SYBR Green (Molecular Probes, Cat#S-7563)

5 % DMSO

Tubes and lids were purchased from BioRad (Cat# TCS0803 and TLS0851)

Cycling parameters were:

95°C for 5 min,

95°C for 10 sec

65°C for 20 sec

72°C for 20 sec

Plate read

80°C for 1 sec

Plate read

Cycle 39 times

Melting curve from 65°C to 95°C, read every 0.2°C, hold 2 sec

Reactions were set up manually in a designated room using designated equipment. qPCRs were performed with the Opticon2 System from MJ Research.

*Taq was isolated as described in F. G. Pluthero (1993) *NAR*21:4850-4851

| **qPCR VALIDATION** |
| --- |
| Evidence of optimisation (from gradients) |
| Specificity (gel, sequence, melt, or digest) |
| For SYBR Green I, Cq of the NTC |
| Standard curves with slope and y-intercept |
| PCR efficiency calculated from slope |
| Confidence interval for PCR efficiency or standard error |
| r2 of standard curve |
| Linear dynamic range |
| Cq variation at lower limit |
| Confidence intervals throughout range |
| Evidence for limit of detection |
| If multiplex, efficiency and LOD of each assay. |

The specificity of the amplification products have been confirmed by size estimations on a 2% agarose gel, sequencing of the products and by analyzing their melting curves. Without a template, no Cq could be determined since it never passed the threshold line. Up to 3% of the uninduced condition was analyzed during efficiency determination.

|  | **length (bp)** | **slope** | **y-intercept** | **% efficiency** | **r2** |
| --- | --- | --- | --- | --- | --- |
| **123019** | 113 | -0.97 | 34.27 | 104 | 0.9392 |
| **117458** | 106 | -1.02 | 33.16 | 98 | 0.8428 |
| **105568** | 86 | -1.01 | 27.62 | 99 | 0.7887 |
| **122261** | 136 | -0.99 | 27.35 | 101 | 0.9822 |
| **106748** | 92 | -0.99 | 25.50 | 101 | 0.9910 |
| **195946** | 149 | -1.03 | 25.16 | 96 | 0.9969 |
| **106402** | 126 | -1.01 | 29.54 | 98 | 0.9868 |
| **101629** | 96 | -0.99 | 27.91 | 101 | 0.9950 |
| **143868** | 104 | -1.04 | 27.66 | 95 | 0.9966 |
| **73360** | 127 | -0.98 | 29.03 | 103 | 0.9977 |

| **DATA ANALYSIS** |
| --- |
| qPCR analysis program (source, version) |
| Cq method determination |
| Outlier identification and disposition |
| Results of NTCs |
| Justification of number and choice of reference genes |
| Description of normalisation method |
| Number and concordance of biological replicates |
| Number and stage (RT or qPCR) of technical replicates |
| Repeatability (intra-assay variation) |
| Reproducibility (inter-assay variation, %CV) |
| Power analysis |
| Statistical methods for result significance |
| Software (source, version) |
| Cq or raw data submission using RDML |

qPCR analysis program (source, version): Biorad, Opticon 3

Cq’s were determined by setting the threshold to -1.0 using a log scale,

No data have been exclude from the calculations

Results of NTCs: no amplification products present thus no Cqs

Justification of number and choice of reference genes: cDNAs had previously been tested with

another reference gene (*UBI2*) with the same results

Description of normalisation method: endogenous reference gene

Number and concordance of biological replicates: 3

Number and stage (RT or qPCR) of technical replicates: 3 at qPCR level, 2 for RT (Mn samples)

Repeatability (intra-assay variation): was below one Cq
